# Amino acid metabolic regulatory network in tumor immune escape: mechanistic exploration and intervention directions

**DOI:** 10.3389/fimmu.2026.1871598

**Published:** 2026-07-31

**Authors:** Zhaoxia Liu, Lin He, Yu Tian, Shipin Feng, Xiongtao Yang, Hui Wu, Qiaoshuang Pu, Yan Wang, Xiaoli Yuan

**Affiliations:** 1 Department of General Internal Medicine, Sichuan Clinical Research Center for Cancer, Sichuan Cancer Hospital &Institute, Sichuan Cancer Center, University of Electronic Science and Technology of China, Chengdu, China; 2 Department of Medical Oncology, Huizhou Central People’s Hospital, Huizhou, Guangdong, China; 3 Department of Pediatric Nephrology, Chengdu Women ‘ s and Children ‘ s Central Hospital, School of Medicine, University of Electronic Science and Technology of China, Chengdu, China

**Keywords:** amino acid metabolism, immunometabolism, immunotherapy resistance, tumor immune escape, tumor microenvironment

## Abstract

Tumor immune escape is not determined solely by immune checkpoints, suppressive cytokines, or changes in immune-cell composition; it is also organized by the metabolic architecture of the tumor microenvironment. Reprogrammed amino acid metabolism contributes to this process by redistributing nutrients, generating immunoregulatory metabolites, and reshaping immune-cell states. Rather than viewing individual amino acid pathways as independent mechanisms, this review proposes a network framework in which nutrient competition, metabolite-mediated communication, and immune-state stabilization are mechanistically coupled across tumor, immune, stromal, vascular, and microbiota-associated compartments. We define four properties of this network: cross-pathway convergence on shared immune outcomes, division of metabolic labor among cell populations, spatial and temporal context dependence, and compensatory feedback that sustains immune suppression. Within this framework, we examine how tryptophan, glutamine, methionine, arginine, and emerging pathways such as asparagine regulate T-cell dysfunction, regulatory T-cell persistence, myeloid polarization, dendritic-cell impairment, and resistance to immune checkpoint blockade. We further discuss how network-level understanding may improve biomarker development and guide cell-selective, temporally optimized, and rational combination strategies. Conceptualizing amino acid metabolism as an interconnected regulatory system may better explain the context-dependent effects of metabolic interventions and support precision immunometabolic therapy.

## Highlights

- Reprogrammed amino acid metabolism is a central driver of tumor immune escape through nutrient competition, metabolite signaling, and immune-cell rewiring.
- Amino acids function not only as metabolic substrates but also as immunoregulatory cues that shape CD8+ T-cell dysfunction, Treg persistence, and myeloid suppression.
- Targeting amino acid metabolic networks may improve biomarker discovery and enhance the efficacy of combination cancer immunotherapy.

## 1 Introduction

Tumor immune escape has traditionally been interpreted through immune checkpoints, suppressive cytokines, defective antigen presentation, and the accumulation of immunoregulatory cell populations (1–4). Although these mechanisms are central, they do not fully explain the persistence and context dependence of immune dysfunction in the tumor microenvironment. Increasing evidence indicates that tumor immune escape is also fundamentally a metabolic problem (5–7). Beyond genetic and signaling alterations, tumor cells actively reshape the local biochemical milieu in ways that profoundly affect immune cell fitness and function. Among the major metabolic features of the tumor microenvironment, amino acid metabolism has emerged as a critical determinant of antitumor immunity (8–10). Tumor cells do not merely adapt to nutrient limitation; rather, they actively reprogram amino acid uptake, synthesis, catabolism, and metabolite release to create a microenvironment that supports tumor survival while restricting immune effector activity. By increasing transporter expression, rewiring metabolic enzymes, and redirecting metabolic flux, cancer cells gain preferential access to key nutrients and generate immunosuppressive metabolites. As a result, amino acid availability in tumors is not simply reduced, but selectively reorganized. This metabolic asymmetry impairs T-cell activation and persistence, weakens dendritic-cell priming capacity, and favors suppressive myeloid programs, thereby reinforcing immune escape even in tumors with immune infiltration (11–14).

Amino acids have classically been regarded as substrates for protein synthesis, nucleotide production, redox balance, and biomass accumulation (15–18). In cancer immunity, however, they are more than passive nutrients. Amino acids function as immunoregulatory signals that shape immune-cell activation, differentiation, and effector capacity. In T cells, amino acid availability is tightly linked to metabolic reprogramming, mTOR signaling, epigenetic remodeling, cytokine production, and cytotoxic function, whereas amino acid restriction promotes proliferative defects and exhaustion-like states (19–22). Similar metabolic sensitivity is observed in macrophages, dendritic cells, and regulatory T cells, indicating that amino acid availability helps determine immune-cell fate across both lymphoid and myeloid compartments. This conclusion is supported by multiple experimental systems rather than by correlative observations alone. *In vitro* T-cell differentiation models have shown that glutamine metabolism can differentially regulate Th1, Th17, cytotoxic T lymphocyte, and regulatory T-cell differentiation through glutaminase-dependent metabolic and chromatin-associated mechanisms (23, 24). Tumor-bearing mouse models have further demonstrated that glutamine blockade can suppress tumor-cell oxidative and glycolytic metabolism while improving the metabolic conditions that support antitumor T-cell activity (25). In addition, studies of methionine competition have shown that tumor cells can outcompete CD8+ T cells for methionine, reduce intracellular S-adenosylmethionine availability, decrease H3K79me2, impair STAT5 signaling, and weaken T-cell antitumor immunity (26). These studies illustrate that amino acid metabolism affects immune cells through experimentally defined mechanisms involving nutrient transport, metabolic flux, mitochondrial function, chromatin regulation, and transcriptional control (27). Equally important, amino acid metabolism generates bioactive intermediates that act as signaling molecules in their own right. Metabolites derived from tryptophan, arginine, methionine, and other amino acids can regulate receptor activation, transcriptional programs, chromatin state, and intercellular communication (28). Thus, the immunological relevance of amino acids depends not only on their abundance, but also on how they are metabolized, by which cells, and into which downstream products.

Previous reviews have commonly organized amino acid metabolism according to individual nutrients, metabolic enzymes, or immune-cell populations (15, 29). Although this approach provides valuable pathway-specific detail, it does not fully explain why distinct metabolic abnormalities frequently produce overlapping immune phenotypes, why metabolic interventions show context-dependent effects, or why inhibition of a single pathway often fails to produce durable immune restoration. Here, we propose that tumor-associated amino acid metabolism should be understood as a multicellular regulatory network rather than as a set of parallel biochemical routes. In this framework, amino acids and their derivatives are connected through shared transport systems, nutrient-sensing pathways, metabolic cofactors, transcriptional and epigenetic regulators, and reciprocal interactions among tumor, immune, stromal, vascular, and microbial compartments. The central conceptual advance of this review is therefore to integrate four network properties: cross-pathway convergence on common immune outcomes, division of metabolic labor among cell populations, spatial and temporal heterogeneity, and compensatory feedback that stabilizes immune escape. Individual amino acid axes are discussed as mechanistic components of this broader network rather than as independent suppressive pathways. This perspective also provides a rationale for biomarker-guided, cell-selective, and combination metabolic interventions. Importantly, the evidence supporting individual components of this network is not equally mature. Some mechanisms, including IDO-mediated tryptophan catabolism, ARG1-associated arginine depletion, and the dependence of activated T cells on glutamine, have been reproduced across multiple experimental systems. Other mechanisms, such as tumor–T-cell methionine competition and its epigenetic consequences, are mechanistically compelling but remain dependent on tumor type and cellular context. By contrast, asparagine-associated immune signaling, microbiota-derived amino acid regulation, and several proposed cross-pathway feedback circuits remain emerging concepts supported by relatively limited experimental evidence. Throughout this review, we therefore distinguish established mechanisms from context-dependent findings and hypothesis-generating models, while identifying the major limitations that currently restrict clinical translation.

## 2 Conceptual framework of amino acid metabolic control in tumor immune escape

### 2.1 Nutrient availability

A central feature of amino acid metabolic control in tumor immune escape is the unequal distribution of nutrients within the tumor microenvironment. Amino acid availability is not determined simply by systemic supply, but by local competition, cellular uptake capacity, metabolic priorities, and tissue organization (15, 30). Tumor cells frequently acquire a strong advantage in this competition through upregulation of amino acid transporters, activation of anabolic programs, and rewiring of catabolic pathways that enable rapid nutrient capture and retention. As a result, the tumor microenvironment becomes a metabolically asymmetric space in which malignant cells maintain privileged access to essential and conditionally essential amino acids, whereas immune effector cells are left in a state of relative deprivation (31, 32). This imbalance has profound implications for antitumor immunity. Activated T cells require substantial amounts of amino acids to support biosynthesis, proliferation, mitochondrial adaptation, and cytokine production. These demands are especially high during sustained antigen exposure, when effector lymphocytes must maintain both rapid cell division and functional competence (33–36). When tumors consume or sequester key amino acids such as glutamine, methionine, arginine, or tryptophan, immune cells are not simply deprived of fuel; they are prevented from executing the metabolic transitions required for effective activation and persistence. In this sense, nutrient restriction becomes a direct mechanism of immune suppression rather than a passive by-product of tumor growth (37, 38). Importantly, nutrient availability within tumors is heterogeneous rather than uniform. Regions of high tumor cell density, poor perfusion, stromal enrichment, or intense myeloid infiltration may each display distinct amino acid landscapes (39, 40). Such spatial heterogeneity means that immune cells entering the tumor do not encounter a single metabolic environment, but rather a series of localized nutrient niches that can differentially shape their fate. Some niches may support transient immune activity, whereas others may favor exhaustion, anergy, or deletion. This spatial dimension is critical because it helps explain why immune infiltrates can be present in tumors yet remain functionally ineffective. Nutrient availability is also influenced by non-malignant cellular components (41, 42). Tumor-associated macrophages, cancer-associated fibroblasts, endothelial cells, and microbiota-related factors can all contribute to amino acid depletion, recycling, or redistribution (15, 43). Accordingly, amino acid restriction in tumors should be understood as a collective property of the tumor ecosystem rather than the consequence of cancer cell metabolism alone. A proper conceptual framework must therefore treat nutrient availability as a dynamic and multicellular determinant of immune competence.

### 2.2 Metabolite signaling

Although nutrient depletion is a major component of amino acid-mediated immune regulation, the immunological consequences of amino acid metabolism cannot be reduced to scarcity alone (44, 45). A second key layer of control lies in metabolite signaling. Once amino acids are taken up and processed, they generate downstream intermediates and end products that can act as biologically active signals. These metabolites influence receptor activity, transcription factor engagement, chromatin organization, redox state, and intercellular communication (8, 46). Consequently, amino acid metabolism exerts immune effects not only by controlling what is absent, but also by generating what is present. This distinction is crucial. In many tumor settings, the suppressive effect of an amino acid pathway is not fully explained by depletion of the parent nutrient. Instead, catabolites themselves actively instruct immune dysfunction. Tryptophan catabolism, for example, does not merely lower tryptophan availability; it also generates metabolites capable of activating transcriptional programs associated with immune tolerance (47–50). Likewise, arginine metabolism can give rise to downstream products that reinforce suppressive myeloid phenotypes, while methionine metabolism influences the supply of methyl donors that regulate gene expression and cell-state stability (51, 52). These examples illustrate that amino acid metabolism is not a simple matter of nutrient consumption, but a system of signal production and interpretation.

Metabolite signaling often acts across cell types. A metabolite generated in tumor cells may reshape macrophage behavior, alter dendritic cell function, or influence T-cell differentiation at a distance. Conversely, metabolites produced by stromal or myeloid cells may feed back onto tumor cells and further strengthen immune resistance (53, 54). This creates a layered communication network in which amino acid-derived molecules function as local immunoregulatory messengers. Through this process, tumors can establish suppressive signaling fields that extend beyond the intracellular metabolic state of any single cell population. Another important aspect of metabolite signaling is duration. Nutrient deprivation may produce acute stress responses, but amino acid-derived metabolites often impose more sustained regulatory effects by altering transcriptional or epigenetic programs. In this way, transient metabolic changes can be converted into relatively stable immune phenotypes, including exhaustion, tolerance, or suppressive myeloid polarization. Therefore, the metabolite signaling layer provides a mechanistic bridge between momentary metabolic disturbance and long-term immune dysfunction.

### 2.3 Cell-state rewiring

Available evidence supports a model in which nutrient competition and metabolite signaling can converge on immune-cell state rewiring. However, the strength of this evidence varies substantially among amino acid pathways and immune-cell populations. This refers to the durable reprogramming of immune cell identity, function, and differentiation in response to the metabolic conditions of the tumor microenvironment (55, 56). Rather than merely causing short-term inhibition, reprogrammed amino acid metabolism can shift immune cells into alternative phenotypic states that are intrinsically less capable of supporting tumor rejection (57, 58). In CD8+ T cells, amino acid restriction and metabolite-mediated suppression can progressively erode effector function and drive the transition toward dysfunctional or exhausted states (59–62). This process involves impaired proliferation, reduced cytokine production, weakened cytotoxicity, altered mitochondrial fitness, and reinforcement of transcriptional programs associated with chronic stimulation and hyporesponsiveness. Importantly, these changes are not solely determined by antigen exposure; they are also shaped by the metabolic conditions under which T cells experience that exposure. Amino acid availability therefore becomes a contextual signal that determines whether persistent antigen recognition results in durable immunity or terminal dysfunction.

In myeloid populations, cell-state rewiring often manifests as stabilization of immunosuppressive phenotypes (63–65). Macrophages exposed to amino acid-derived suppressive cues may adopt transcriptional and metabolic programs associated with tissue remodeling, anti-inflammatory signaling, and promotion of tumor progression (66). Myeloid-derived suppressor cells may further amplify this environment by consuming nutrients, producing inhibitory metabolites, and suppressing lymphocyte activation (67). Dendritic cells, under similar conditions, may lose antigen-presenting efficiency or fail to provide adequate co-stimulatory signals, thereby weakening the initiation of adaptive antitumor responses. Thus, amino acid metabolic rewiring extends across innate and adaptive immunity alike. Regulatory T cells represent a particularly important example of metabolically conditioned cell-state adaptation. Compared with conventional effector T cells, they often display greater resilience in nutrient-poor and suppressive environments, allowing them to preserve immune inhibitory function even when other lymphocyte populations are weakened (68, 69). This relative metabolic advantage further skews the immune balance toward tolerance. As a result, the tumor microenvironment does not simply suppress immunity in general; it selectively rewires immune composition and function in ways that favor tumor persistence. Cell-state rewiring is especially relevant because it helps explain why immune dysfunction in tumors is often self-reinforcing (55, 70). Once suppressive immune populations are established and effector cells become metabolically impaired, these altered states can reshape the microenvironment even further by changing cytokine production, nutrient utilization, and cell-cell signaling. These observations suggest that amino acid metabolic rewiring may generate feedback loops that stabilize immune escape, although most such multicellular feedback circuits have not yet been directly reconstructed or functionally validated *in vivo*.

### 2.4 Major cellular components of the amino acid metabolic network

Amino acid metabolic control in tumors cannot be attributed to a single dominant cell type. Rather, it emerges from coordinated and competitive interactions among multiple cellular populations, each of which both shapes and responds to local nutrient conditions (71). A network-based model must therefore identify the major cellular components of this metabolic ecosystem and define their distinct functional roles.

Tumor cells occupy the central initiating position in this network. Through enhanced transporter expression, biosynthetic flexibility, and catabolic rewiring, they create the metabolic pressures that define the amino acid landscape of the tumor microenvironment. Yet tumor cells are not the only architects of amino acid-mediated immune escape (15, 43). Tumor-associated macrophages are major interpreters and amplifiers of metabolic cues. They respond to amino acid-derived signals by adopting phenotypes that promote tolerance, matrix remodeling, angiogenesis, and immune suppression, while also contributing to local nutrient consumption and metabolite release (72–76).

Myeloid-derived suppressor cells represent another important component. These cells are metabolically active suppressors that not only inhibit T-cell function directly but also reshape the amino acid milieu through consumption and enzymatic processing. Dendritic cells, although often discussed primarily in the context of antigen presentation, are also metabolically vulnerable cells whose priming capacity is strongly influenced by amino acid sufficiency and stress signaling (77–79). Their dysfunction can therefore be viewed as a metabolically mediated failure of immune initiation. Effector T cells are among the most important targets of the amino acid metabolic network (80, 81). Their function depends on rapid and coordinated metabolic reprogramming, making them especially sensitive to nutrient deprivation and suppressive metabolites. Regulatory T cells, in contrast, may exploit or tolerate these conditions more effectively, thereby acquiring a relative advantage in the tumor microenvironment (82, 83). This differential sensitivity between effector and suppressive lymphocyte populations is a key mechanism through which amino acid metabolism reshapes immune balance. Other stromal and environmental elements also deserve consideration. Cancer-associated fibroblasts can influence nutrient accessibility and metabolic compartmentalization through extracellular matrix remodeling, metabolite exchange, and paracrine signaling. Endothelial cells contribute to the spatial regulation of nutrient delivery through vascular architecture and perfusion status (84, 85). In some settings, microbiota-derived metabolites or local microbial communities further modulate amino acid-associated immune pathways, particularly those involving tryptophan catabolism and aryl hydrocarbon receptor signaling. These additional layers reinforce the idea that amino acid metabolism in tumors is an ecosystem property rather than a tumor-cell-autonomous event.

**Figure 1:**
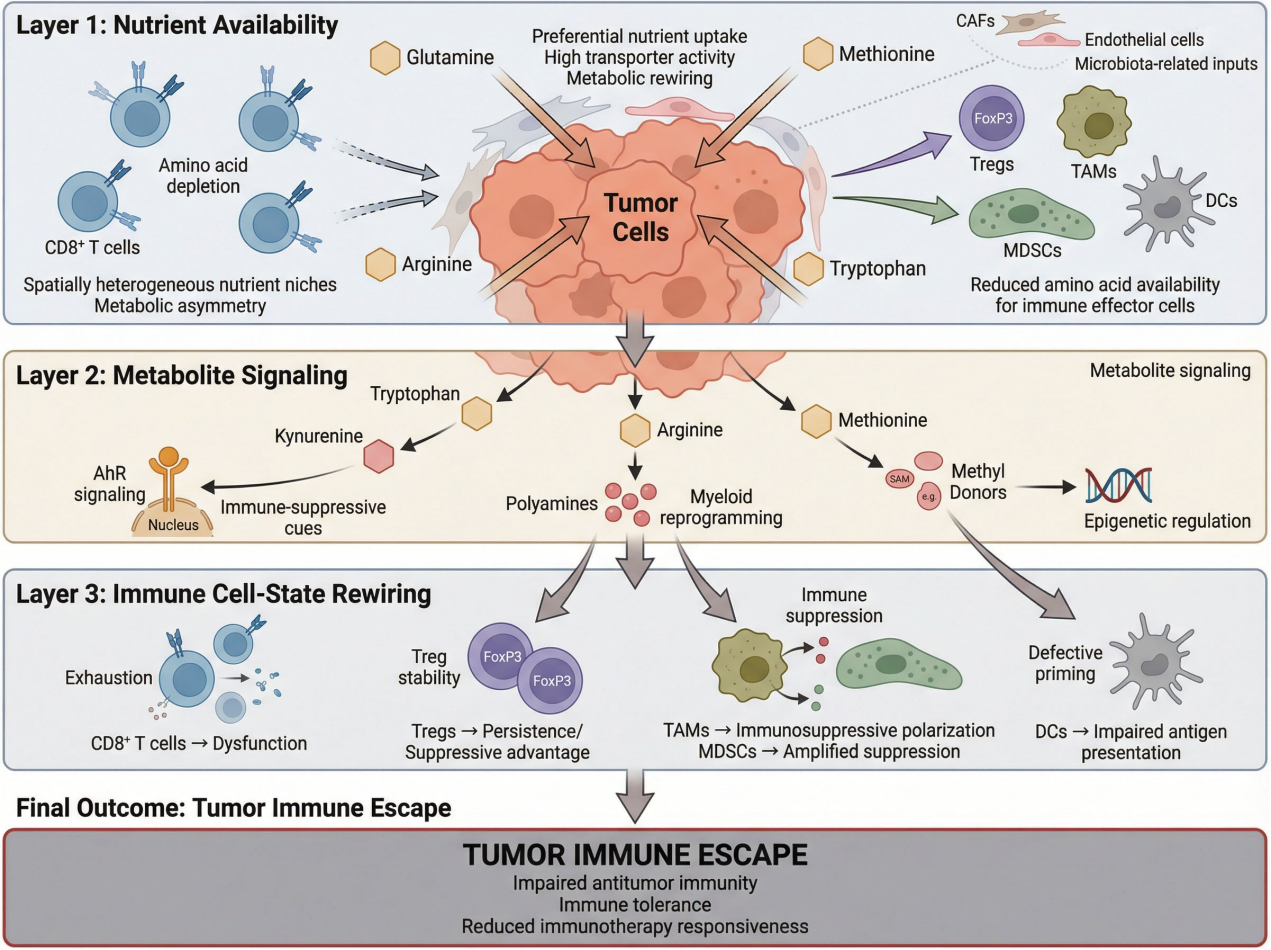
Conceptual framework of amino acid metabolic control in tumor immune escape. Tumor cells reshape amino acid availability through preferential nutrient uptake and metabolic rewiring, generating a metabolically asymmetric microenvironment. In parallel, amino acid-derived metabolites function as immunoregulatory signals that reprogram immune-cell states, including CD8+ T-cell exhaustion, Treg persistence, suppressive myeloid polarization, and dendritic-cell dysfunction, ultimately promoting tumor immune escape.

Taken together, these cellular components form a dynamic network in which nutrient competition, metabolite exchange, and phenotypic rewiring are tightly intertwined. Understanding tumor immune escape therefore requires moving beyond the study of isolated pathways in isolated cells. Instead, amino acid metabolism should be viewed as a multicellular regulatory architecture that governs how resources are distributed, how signals are interpreted, and how immune states are stabilized within tumors. **Figure 1** summarizes the conceptual framework by which reprogrammed amino acid metabolism drives tumor immune escape. It highlights three interconnected layers—nutrient competition, metabolite signaling, and immune-cell state rewiring—that together establish an immunosuppressive tumor microenvironment.

## 3 Nutrient competition in the tumor microenvironment

### 3.1 Tumor–immune competition for metabolic resources

Tumor and immune cells compete directly for amino acids, but malignant cells frequently gain an advantage through enhanced transporter expression, anabolic flexibility, and adaptation to poorly perfused tumor niches (90–94). This unequal access restricts the biosynthetic, mitochondrial, and epigenetic programs required for immune-cell activation. Because effector CD8+ T cells have particularly high anabolic demands, amino acid competition preferentially weakens tumoricidal immunity while allowing metabolically adaptable Tregs and suppressive myeloid populations to persist.

The importance of nutrient competition lies partly in its selectivity. Different immune cell subsets exhibit distinct metabolic dependencies and differ in their capacity to tolerate nutrient stress. Effector CD8+ T cells are highly vulnerable to deprivation because they must maintain sustained anabolic activity under conditions of chronic antigen stimulation (86, 87). By contrast, certain suppressive populations, including regulatory T cells and some myeloid subsets, may better adapt to limited nutrient environments. This means that competition for amino acids does not simply suppress all immunity uniformly. Rather, it restructures the immune landscape in a biased manner, preferentially weakening tumoricidal responses while preserving or even enriching suppressive cell states. The outcome is a metabolically stratified ecosystem in which nutrient access becomes a mechanism of immune selection. Nutrient competition is also temporally dynamic. Early in tumor development, local amino acid restriction may primarily affect immune priming or infiltration (88, 89). As tumors progress, prolonged competition can generate a chronic state of metabolic insufficiency that drives immune dysfunction more deeply into the tissue. Meanwhile, therapeutic interventions such as chemotherapy, radiotherapy, targeted therapy, or immune checkpoint blockade can further alter nutrient flux, vascular integrity, and stromal composition, thereby reshaping amino acid competition in ways that may either support or hinder antitumor responses. For these reasons, nutrient competition should be understood not as a static hallmark, but as a dynamic and therapeutically relevant process that evolves during tumor progression and treatment.

### 3.2 Glutamine metabolism in T-cell differentiation, mitochondrial fitness, and effector dysfunction

Glutamine is a central metabolic substrate for activated T cells because it provides both carbon and nitrogen to support nucleotide synthesis, amino acid biosynthesis, redox balance, and mitochondrial anaplerosis (90). After T-cell receptor stimulation, T cells markedly increase glutamine uptake and glutaminolysis to meet the bioenergetic and biosynthetic demands of clonal expansion. Through conversion into glutamate and α-ketoglutarate, glutamine replenishes the tricarboxylic acid cycle and supports mitochondrial respiration, ATP production, and metabolic intermediates required for effector differentiation (25). Therefore, glutamine availability directly influences whether activated T cells acquire a productive effector phenotype or enter a metabolically compromised state.

Glutamine metabolism also participates in T-cell fate decisions. GLS-dependent glutaminolysis can differentially regulate Th1, Th17, cytotoxic T lymphocyte, and regulatory T-cell differentiation by controlling mTOR activity, α-ketoglutarate availability, redox balance, and epigenetic remodeling (91, 92). The requirement for glutamine during T-cell activation is well supported, but its influence on T-cell differentiation is not uniform. Genetic or pharmacological inhibition of glutaminolysis has produced different effects across CD4+ and CD8+ T-cell subsets, depending on activation conditions, cytokine exposure, nutrient availability, and the duration of metabolic perturbation. Therefore, evidence that glutamine supports lymphocyte biosynthetic and mitochondrial fitness should be distinguished from the more context-dependent conclusion that glutamine restriction consistently favors regulatory or dysfunctional T-cell states. These findings indicate that glutamine is not only a growth-supporting nutrient but also a fate-instructive metabolite for T cells. At the molecular level, glutamine-dependent T-cell activation is coordinated by nutrient transporters, metabolic enzymes, and signaling sensors (93, 94). TCR and co-stimulatory signaling increase the expression of glutamine transporters such as SLC1A5/ASCT2 and SLC7A5/CD98, while c-Myc promotes anabolic metabolic reprogramming and supports glutamine uptake, glycolysis, and biosynthetic gene expression (95, 96). After uptake, glutamine is converted by GLS1 into glutamate and then into α-ketoglutarate, which supports TCA-cycle anaplerosis, mitochondrial respiration, and mTORC1 activity (97, 98). These signals contribute to IL2 transcription, IFN-γ production, granzyme B and perforin expression, and sustained clonal expansion. Conversely, glutamine deprivation or impaired glutaminolysis can activate nutrient-stress responses, weaken mTORC1–c-Myc-dependent effector programming, reduce mitochondrial spare respiratory capacity, increase oxidative stress, and promote exhaustion-like dysfunction in tumor-infiltrating CD8+ T cells.

The mitochondrial consequences of glutamine deprivation are especially relevant in tumors. When tumor cells outcompete T cells for glutamine, CD8+ T cells may fail to maintain TCA-cycle flux, mitochondrial membrane potential, respiratory capacity, and redox homeostasis (99, 100). This mitochondrial stress can promote excessive reactive oxygen species accumulation, apoptotic susceptibility, reduced spare respiratory capacity, and impaired metabolic flexibility. Functionally, these changes are associated with decreased proliferation, weakened cytokine secretion, reduced granzyme and perforin-associated cytotoxicity, and diminished persistence of tumor-infiltrating T cells (101, 102). Thus, glutamine restriction can drive altered T-cell function by linking nutrient competition to mitochondrial dysfunction and exhaustion-like immune impairment. This axis is therapeutically complex. Complete or nonselective glutamine blockade may compromise activated lymphocytes, whereas tumor-selective or context-optimized glutamine targeting may reduce tumor metabolic dominance, relieve hypoxia and acidosis, reprogram suppressive myeloid cells, and indirectly improve antitumor T-cell function. Therefore, glutamine metabolism should be discussed as a bidirectional immunometabolic regulator: it is required for T-cell differentiation and mitochondrial fitness, yet tumor glutamine addiction can deprive T cells of this resource and contribute to immune escape.

### 3.3 Methionine competition, epigenetic fitness, and exhaustion-associated chromatin remodeling in T cells

Methionine competition adds another layer of complexity to amino acid-mediated immune regulation because its effects extend beyond conventional biosynthesis into the realm of epigenetic control. Methionine serves as a precursor for S-adenosylmethionine, the principal methyl donor for DNA, RNA, protein, and histone methylation (103, 104). Through this function, methionine availability directly influences chromatin architecture, transcriptional programming, RNA metabolism, and long-term cell-state stability. In tumors, where methionine uptake and one-carbon metabolic flux are frequently enhanced, competition for methionine can therefore have consequences that are qualitatively different from those produced by general nutrient deprivation.

In T cells, adequate methionine availability is essential for sustaining activation-associated epigenetic remodeling (105, 106). Effective antitumor responses require not only rapid proliferation but also coordinated transcriptional reprogramming that supports effector differentiation, cytokine expression, and memory potential. These processes depend on sufficient methyl donor availability to maintain appropriate chromatin accessibility and gene-regulatory activity. When tumor cells dominate methionine consumption, T cells may experience an epigenetic form of metabolic insufficiency in which their ability to establish and preserve effector programs becomes compromised (107–109). The result is not simply slower growth, but reduced functional plasticity and weakened immune competence. This concept is particularly relevant to T-cell exhaustion. A representative mechanistic study has linked tumor–T-cell methionine competition to specific epigenetic changes in CD8+ T cells. Tumor cells with high methionine transporter activity, particularly SLC43A2, can outcompete T cells for methionine uptake, thereby reducing intracellular methionine and SAM levels in tumor-infiltrating CD8+ T cells (26). Reduced SAM availability limits methyltransferase-dependent histone methylation, with H3K79me2 representing a well-characterized affected mark. Loss of H3K79me2 at effector-associated loci can decrease STAT5 expression and p-STAT5 signaling, thereby weakening IL2-, IFNG-, GZMB-, and cytotoxicity-associated transcriptional programs (26). In addition to histone methylation, early methionine insufficiency may alter protein methylation-dependent signaling, including KCa3.1-associated regulation of Ca²^+^–NFAT1 activity. Limited evidence further suggests that methionine-sensitive protein methylation may influence KCa3.1-associated Ca2+–NFAT signaling. However, the direct relevance of this mechanism to tumor-infiltrating T-cell exhaustion remains insufficiently established and should currently be regarded as hypothesis-generating (110). Thus, methionine restriction promotes T-cell dysfunction through defined epigenetic and signaling events, including SAM depletion, H3K79me2 loss, STAT5 impairment, and NFAT1-associated exhaustion programming.

Chronic antigen exposure in tumors already predisposes T cells to dysfunctional transcriptional states, and methionine restriction may further reinforce this trajectory by limiting the methylation-dependent mechanisms needed to preserve effector identity (111). Thus, methionine competition can contribute to immune escape by shifting the epigenetic balance of T cells away from durable antitumor function and toward progressive dysfunction. This provides an important conceptual bridge between amino acid availability and long-term immune cell fate. Methionine competition also illustrates why amino acid-mediated immune escape cannot be understood solely through short-term metabolic readouts (112, 113). Because methionine influences the epigenetic machinery, its effects may persist even after nutrient conditions change. Transient restrictions in methionine access may leave durable molecular imprints on immune cells, altering their responsiveness to later stimulation or immunotherapy. This makes the methionine axis especially relevant when considering therapeutic timing, reversibility of immune dysfunction, and the design of combination strategies aimed at restoring immune competence.

Recent evidence further suggests that methionine availability can influence exhaustion-associated chromatin states in tumor-infiltrating CD8+ T cells. Because methionine supplies S-adenosylmethionine for methylation reactions, methionine restriction may reduce the methylation capacity required to maintain effector-associated chromatin accessibility and transcriptional competence (114, 115). Tumor cells with enhanced methionine uptake or methionine-cycle activity can thereby deprive T cells of methyl donor availability, leading to altered histone methylation, reduced expression of effector-associated transcriptional programs, and increased vulnerability to exhaustion (116, 117). In this context, T-cell exhaustion is not only driven by persistent antigen stimulation and inhibitory receptor signaling, but also by a metabolically imposed epigenetic constraint. Methionine insufficiency may help lock tumor-infiltrating T cells into exhaustion-associated chromatin configurations, characterized by reduced accessibility of effector loci and reinforcement of dysfunctional transcriptional modules. Therefore, methionine competition provides a mechanistic link between amino acid availability, chromatin remodeling, and the durability of T-cell dysfunction in tumors.

### 3.4 Arginine depletion and impaired immune surveillance

Arginine is another major determinant of antitumor immunity and has long been associated with immune regulation in cancer. It supports T-cell proliferation, survival, activation, and memory formation, and its availability can strongly influence the capacity of lymphocytes to maintain effective responses under conditions of chronic stimulation (118, 119). Within tumors, however, arginine is frequently depleted through consumption and enzymatic processing by malignant cells and suppressive myeloid populations. This creates a local environment in which immune surveillance is metabolically constrained. The consequences of arginine depletion are especially evident in T cells. Reduced arginine availability impairs cell-cycle progression, limits biosynthetic readiness, and dampens cytokine production, thereby compromising the expansion and function of tumor-reactive lymphocytes (120–123). Moreover, arginine insufficiency may favor the emergence of dysfunctional immune states by weakening the metabolic support required for sustained effector responses. In this way, arginine depletion acts not only as a barrier to acute activation but also as a chronic force that undermines long-term immune competence.

Arginine metabolism is particularly important because of its strong connection to suppressive myeloid biology. Tumor-associated macrophages and related myeloid populations can metabolize arginine in ways that both reduce its availability to T cells and generate downstream products supportive of immune suppression, tissue remodeling, and tumor progression. Thus, arginine is a node where nutrient competition and metabolite signaling closely intersect. Depletion of the parent amino acid and generation of suppressive derivatives are often biologically coupled, amplifying the overall impact of this pathway on tumor immune escape (123, 124). From a conceptual standpoint, the arginine axis demonstrates that nutrient competition is rarely neutral in direction or consequence. It is frequently organized by suppressive cellular programs that deliberately align resource deprivation with the strengthening of immune-inhibitory cell states. This duality helps explain why arginine-related pathways remain attractive yet complex therapeutic targets. Interventions aimed at restoring arginine availability or blocking its suppressive processing may improve antitumor immunity, but they must be calibrated carefully to account for the multiple cell types that participate in arginine turnover within tumors.

### 3.5 Stromal and microbial contributions to amino acid deprivation

Although nutrient competition is often described as a direct contest between tumor cells and immune cells, this binary model is incomplete. The tumor microenvironment includes a broader set of stromal and environmental components that substantially influence amino acid accessibility, distribution, and turnover (118, 125). Among these, cancer-associated fibroblasts, endothelial abnormalities, extracellular matrix remodeling, and microbiota-related factors all contribute to the construction of local amino acid niches. As a result, amino acid deprivation within tumors should be understood as an ecosystem-level phenomenon rather than a tumor-cell-autonomous event.

Cancer-associated fibroblasts can affect amino acid availability in several ways (126–129). By remodeling the extracellular matrix and altering tissue architecture, they influence diffusion barriers, cellular proximity, and spatial nutrient gradients (130, 131). They may also participate in metabolite exchange or support tumor cell metabolic adaptation through paracrine signaling and substrate provisioning. These stromal effects can intensify nutrient restriction in ways that selectively disadvantage immune cells attempting to infiltrate or function within tumor tissue. Similarly, aberrant vascular structure and perfusion reduce efficient nutrient delivery, creating poorly nourished microregions in which immune cells face additional metabolic stress beyond direct competition with tumor cells (132). Microbiota-related influences add a further dimension to this landscape. Representative studies have begun to define the biological contexts in which microbial metabolites influence antitumor immunity. For example, work in checkpoint blockade models showed that intestinal Bifidobacterium pseudolongum enhanced immunotherapy response through production of the purine metabolite inosine, which acted on T cells under defined co-stimulatory conditions (133). Although inosine is not itself an amino acid catabolite, this study provides an important experimental paradigm showing how microbiota-derived metabolites can alter tumor immunity *in vivo*. In parallel, microbial tryptophan-derived indoles and related metabolites can engage AhR-dependent immune pathways, suggesting that microbiota-associated regulation of tryptophan metabolism may be particularly relevant in mucosal or gastrointestinal tumor settings. These findings indicate that microbiota-linked amino acid metabolism should be discussed in a context-specific manner, including the microbial species, metabolite class, tumor location, immune-cell target, and treatment setting. In some tumor settings, microbial communities or microbiota-derived metabolites interact directly with amino acid pathways, particularly those involving tryptophan metabolism and immune receptor signaling (134). Local or tissue-associated microbes may consume amino acids, generate immunoregulatory catabolites, or alter the activation state of myeloid cells in ways that indirectly influence nutrient competition. These interactions broaden the concept of amino acid regulation from an intratumoral metabolic issue to a host–tumor–microenvironmental interface involving external metabolic inputs.

**Figure 2:**
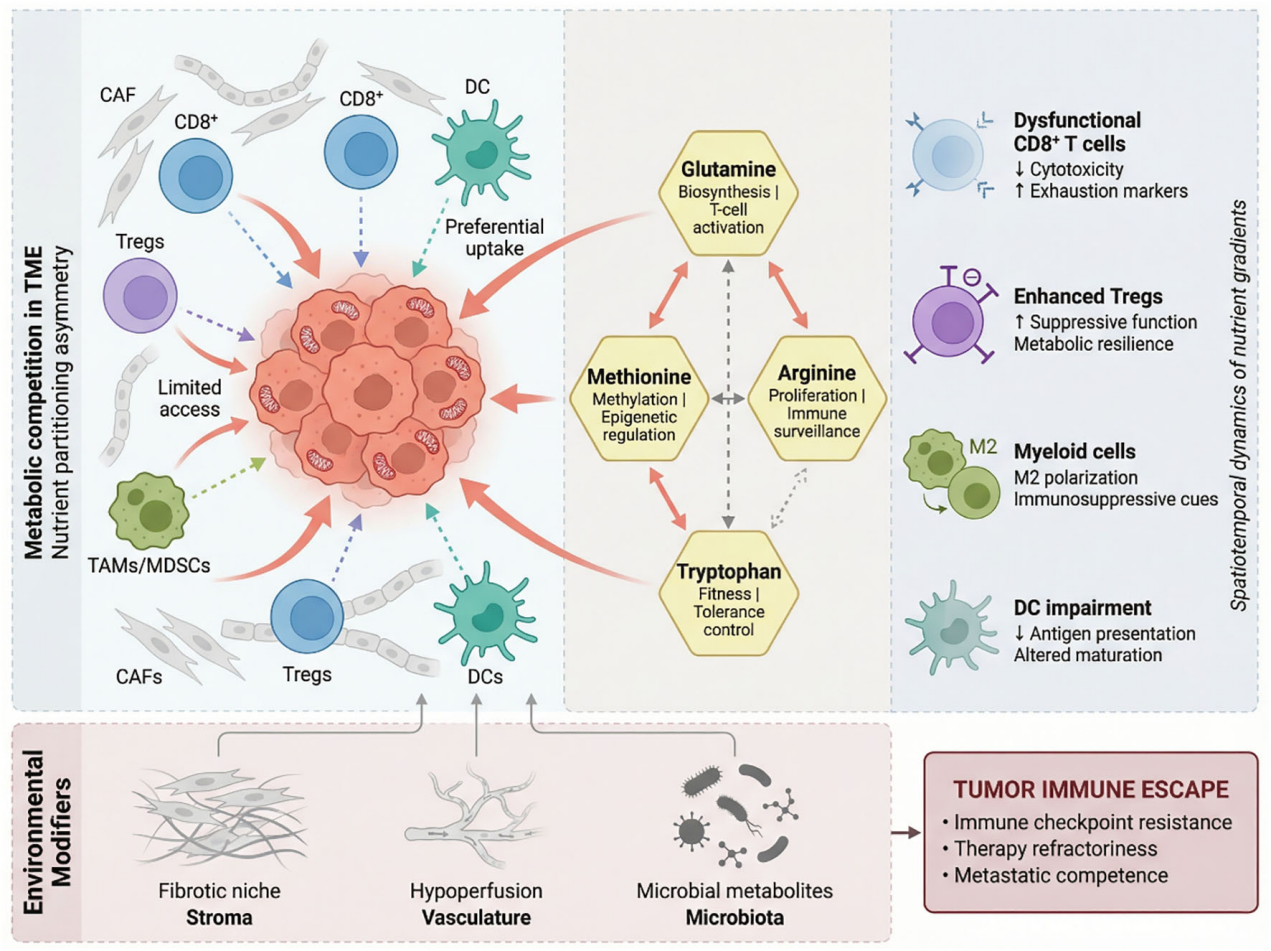
Nutrient competition in the tumor microenvironment. Tumor cells preferentially consume key amino acids, including glutamine, methionine, arginine, and tryptophan, thereby creating a metabolically asymmetric microenvironment. This nutrient deprivation selectively impairs effector immune cells, favors suppressive immune states, and is further shaped by stromal and microbial influences, ultimately contributing to tumor immune escape.

The inclusion of stromal and microbial contributors is important because it shifts the interpretation of amino acid deprivation away from simple scarcity and toward organized metabolic governance (135, 136). Nutrient conditions in tumors are not accidental consequences of rapid cell growth alone. They are shaped by multicellular interactions that define who gains access to metabolic resources, how those resources are transformed, and which cell populations retain functional advantage under stress. This view is essential for understanding why therapeutic approaches targeting amino acid metabolism often produce context-dependent outcomes. The efficacy of such interventions depends not only on tumor cell metabolism, but also on the broader cellular and ecological architecture of the tumor microenvironment. Collectively, tumor, stromal, vascular, and microbial factors generate spatially heterogeneous amino acid niches that selectively impair effector immunity (**Figure 2**).

## 4 Amino acid-derived metabolite signaling in immune suppression

### 4.1 Tryptophan catabolism and kynurenine–AhR signaling

Among amino acid-associated immunoregulatory pathways, tryptophan catabolism remains one of the most intensively studied and conceptually influential (137–139). Its importance lies not only in the depletion of tryptophan itself, but also in the generation of bioactive metabolites that actively instruct immune suppression. In tumors, enhanced conversion of tryptophan through indoleamine 2, 3-dioxygenase 1, indoleamine 2, 3-dioxygenase 2, or tryptophan 2, 3-dioxygenase redirects this essential amino acid away from biosynthetic use and toward kynurenine pathway metabolites. This metabolic shift creates a dual suppressive effect: local tryptophan scarcity limits the metabolic fitness of immune effector cells, while kynurenine and related catabolites act as signaling mediators that reinforce tolerance.

The immunological significance of tryptophan depletion extends beyond simple nutrient shortage. T cells are particularly sensitive to reduced tryptophan availability because adequate amino acid supply is required to sustain activation, proliferation, and cytokine production (140–142). In chronically stimulated tumor settings, this requirement becomes even more critical. When tryptophan is persistently restricted, T cells may fail to maintain productive effector programs, becoming more susceptible to anergy-like dysfunction, reduced cytotoxicity, and progressive exhaustion. Thus, tryptophan deprivation functions as both a metabolic and immunological checkpoint that constrains antitumor responses. However, the suppressive impact of this axis is amplified by the signaling activity of kynurenine. Kynurenine is not merely a degradation product but an active mediator capable of reshaping immune behavior across multiple cell types. A central mechanism involves activation of the aryl hydrocarbon receptor, a ligand-responsive transcription factor that links metabolite sensing to gene regulation. After ligand binding, AhR translocates into the nucleus, dimerizes with ARNT, and binds xenobiotic response elements to regulate both canonical metabolic genes and immune-regulatory programs (143, 144). In this context, canonical AhR targets such as CYP1A1, CYP1B1, and AHRR can serve as markers of pathway activation, whereas immune-related targets and programs, including IDO1 feedback, IL10, TGFB1, FOXP3-associated regulatory programs, and PD-1/PD-L1-related inhibitory signaling, help explain how kynurenine–AhR signaling promotes immune tolerance (145, 146). In myeloid cells, AhR activation can suppress pro-inflammatory antigen-presenting programs while enhancing tolerogenic macrophage and dendritic-cell states. In T cells, this pathway may reduce effector differentiation and favor Treg stability or dysfunctional phenotypes. Therefore, the tryptophan–kynurenine–AhR axis should not be described only as a linear “tryptophan depletion–AhR activation–immune tolerance” pathway, but as a transcriptional regulatory module involving defined AhR target genes and cell-type-specific immunosuppressive outputs.

In macrophages and other myeloid populations, kynurenine–AhR signaling can promote tolerogenic transcriptional programs associated with suppression, tissue remodeling, and support of tumor progression (147, 148). In lymphoid cells, this pathway may further weaken effector differentiation while favoring regulatory or dysfunctional states. As a result, tryptophan catabolism represents a paradigmatic example of how amino acid metabolism couples nutrient loss to metabolite-driven immune rewiring. Importantly, IDO-mediated tryptophan catabolism should not be interpreted solely as a tumor-cell-autonomous pathway. In many tumor microenvironments, myeloid populations, including tumor-associated macrophages, MDSCs, and tolerogenic dendritic cells, represent important sources and responders of IDO activity (47, 149). These cells can reinforce immune tolerance by depleting tryptophan, producing kynurenine, activating AhR-dependent transcriptional programs, and suppressing effector T-cell priming and expansion. Thus, the IDO–kynurenine axis provides a representative example of myeloid cell immunometabolism, in which amino acid catabolism is directly coupled to immune-cell polarization and checkpoint resistance.

The tryptophan–kynurenine–AhR axis is also notable because it connects tumor metabolism with broader host and microenvironmental inputs (150). Microbiota-derived tryptophan metabolites can engage overlapping receptor systems and further influence macrophage polarization, mucosal immunity, and inflammatory tone. This means that tryptophan metabolism in tumors is not solely a tumor-intrinsic pathway, but part of a larger signaling interface that integrates malignant cells, immune populations, stromal components, and microbial factors. Such complexity helps explain the context dependence of tryptophan-targeted interventions and underscores the need to evaluate this pathway in ecological rather than purely enzymatic terms. The biological basis of tryptophan depletion and kynurenine–AhR signaling is supported by extensive preclinical evidence (151, 152). However, the relative contribution of tumor-cell IDO1, myeloid-cell IDO1, TDO2, IL4I1-derived AhR ligands, and microbiota-derived metabolites varies among tumors (153, 154). Moreover, the strength of the mechanistic evidence contrasts with the limited and inconsistent clinical efficacy of broadly applied IDO1 inhibition (155, 156). This discrepancy suggests that pathway expression alone may not identify tumors that are functionally dependent on the kynurenine–AhR axis and highlights the need for pharmacodynamic biomarkers, measurement of pathway activity, and evaluation of compensatory ligand sources.

### 4.2 Methionine cycle metabolites and methylation-dependent immune regulation

Methionine metabolism occupies a distinct position within amino acid-mediated immune control because its downstream consequences are closely linked to methylation-dependent regulation of gene expression and cell-state stability (157–159). Once methionine enters the methionine cycle, it contributes to the production of S-adenosylmethionine, the universal methyl donor used in DNA, RNA, histone, and protein methylation reactions. Through this role, methionine metabolism becomes a critical interface between nutrient availability and epigenetic governance. In the tumor microenvironment, enhanced methionine uptake and one-carbon metabolic reprogramming by cancer cells can therefore influence immunity not only by restricting methionine supply to immune cells, but also by altering the methyl donor landscape that determines how cellular programs are executed and maintained (160). This layer of regulation is highly relevant to T-cell biology. Effective T-cell activation requires rapid transcriptional remodeling and sustained expression of effector genes, processes that depend on properly coordinated methylation dynamics. When methionine availability is reduced, or when methyl donor balance is otherwise disrupted, immune cells may lose the epigenetic support needed to preserve effector identity and functional plasticity. In tumors, such restriction can reinforce dysfunctional transcriptional states and weaken the capacity of T cells to sustain antitumor activity. In this sense, methionine cycle metabolites serve as molecular translators that convert metabolic conditions into stable gene-regulatory consequences (161, 162). Methionine-dependent immune regulation is also relevant beyond T cells. Altered methyl donor availability can affect innate immune sensing, inflammatory responsiveness, and the expression of immune checkpoints or suppressive mediators in tumor and immune cells alike. These effects broaden the significance of methionine metabolism from a simple issue of amino acid competition to a mechanism of multilayered immune control. Notably, methionine restriction may have context-dependent consequences. In some settings, it may disrupt tumor-promoting epigenetic programs and enhance antitumor immunity, for example by relieving repression of innate immune pathways. In other contexts, excessive sulfur or methyl donor limitation may impair immune competence, particularly when immune cells themselves become metabolically compromised. This duality makes the methionine axis especially important for translational discussion, because it illustrates that amino acid-directed therapy can either restore or undermine immunity depending on timing, dosage, and cellular specificity. At the mechanistic level, the methionine cycle regulates immune-cell fate through several methylation-dependent layers (115, 163). SAM availability determines the activity of histone and protein methyltransferases, whereas changes in the SAM/SAH balance can alter the global methylation potential of T cells (117, 164). In tumor-infiltrating CD8+ T cells, reduced methionine availability may impair histone methylation marks associated with effector gene expression, thereby weakening STAT5-dependent cytotoxic programs and favoring dysfunctional transcriptional remodeling. In addition, altered protein methylation may influence signaling molecules that regulate calcium–NFAT activity, providing another route through which methionine insufficiency promotes exhaustion-associated gene expression. These findings indicate that methionine metabolism should be interpreted as an epigenetic regulator of T-cell fate rather than simply as a nutrient-supporting pathway.

Conceptually, methionine cycle metabolites show how amino acid-derived signaling can operate through relatively durable molecular mechanisms. Unlike transient signaling molecules that rapidly fluctuate, methyl donor-dependent regulation may leave longer-lasting imprints on cell identity, responsiveness, and differentiation. This gives methionine metabolism a powerful role in stabilizing either effective immunity or chronic dysfunction within tumors.

### 4.3 Arginine-derived polyamines and myeloid suppression

Arginine metabolism represents another major node where amino acid-derived metabolites actively shape the suppressive tumor microenvironment. Although arginine depletion itself impairs T-cell activation and proliferation, the downstream products of arginine metabolism add an additional layer of immunoregulatory complexity (165, 166). Of particular importance are polyamines, a class of small cationic molecules involved in chromatin organization, cellular proliferation, stress adaptation, and immune regulation. In tumors, polyamine accumulation is frequently associated with growth advantage and immunosuppressive tissue remodeling. The arginine axis has a regulatory logic distinct from the tryptophan and methionine pathways (167–169). ARG1 converts arginine into ornithine, which can be further metabolized by ornithine decarboxylase 1 to generate putrescine, spermidine, and spermine (170, 171). These polyamines support suppressive myeloid-cell survival, stress adaptation, chromatin organization, and tissue-remodeling programs (172, 173). At the same time, arginine depletion can reduce TCR-associated signaling competence and impair T-cell proliferation (174, 175). Thus, arginine metabolism suppresses antitumor immunity through two coordinated mechanisms: depletion of a T-cell-supportive amino acid and production of polyamine-mediated myeloid suppressive signals.

The relevance of polyamines to immune escape lies largely in their effects on myeloid cells, especially tumor-associated macrophages. Preclinical studies suggest that polyamines can influence macrophage differentiation and support tissue-remodeling or immunoregulatory phenotypes. However, the extent to which polyamines directly stabilize tumor-associated macrophage states *in vivo*, independently of other arginine-derived signals, remains incompletely defined (176). Polyamine-rich conditions have been associated with transcriptional and metabolic programs related to tissue repair and immune regulation, but these effects vary with macrophage origin, activation state, tumor model, and the specific polyamine examined. In this way, arginine metabolism helps create a myeloid compartment that not only suppresses lymphocyte function directly but also maintains a microenvironment conducive to tumor persistence (177). This pathway is conceptually important because it illustrates how tumors can convert a contested nutrient into a suppressive communication system. Arginine consumed by tumor cells or processed within the tumor ecosystem does not simply disappear from immune use; it can be redirected into metabolites that actively strengthen the very cell states that sustain immune evasion (178). Thus, the arginine axis exemplifies a broader principle of tumor immunometabolism: nutrients are not merely lost to the immune system, but are often transformed into signals that deepen the suppressive architecture of the microenvironment. Arginine-derived polyamine signaling also reinforces the need for multicellular analysis. The suppressive consequences of this pathway depend not only on the biochemical machinery of tumor cells, but also on metabolite transfer, myeloid uptake, and tissue-contextual interpretation of polyamine exposure. As a result, targeting polyamine-related immune suppression may require strategies that go beyond simple inhibition of one enzyme, instead accounting for the distribution of metabolic labor across tumor and stromal compartments. Accordingly, ARG1-mediated arginine depletion should be regarded as a comparatively well-established mechanism of myeloid suppression, whereas the contribution of individual downstream polyamines to macrophage state stabilization remains more context dependent.

### 4.4 Asparagine and emerging signaling axes in tumor immune escape

Compared with tryptophan, methionine, glutamine, or arginine, asparagine has historically received less attention in discussions of tumor immune escape. Yet recent work suggests that asparagine may represent an important emerging axis in amino acid-centered immune regulation (179, 180). This is significant because it expands the field beyond the classical pathways that have dominated cancer immunometabolism and highlights the possibility that additional amino acids serve as context-dependent signaling regulators rather than merely biosynthetic substrates.

Asparagine appears to influence tumor immunity through mechanisms that extend beyond its role in protein synthesis and nitrogen exchange. Emerging evidence suggests that altered asparagine metabolism can intersect with innate immune sensing pathways and shape the stability or activity of molecules involved in type I interferon signaling (179). Because type I interferon responses are crucial for antitumor immune priming, dendritic cell activation, and sensitivity to immunotherapy, disruption of this axis may provide tumors with a potent route to immune escape. In this framework, asparagine becomes not simply a metabolite supporting tumor survival, but a regulator of the signaling environment that determines whether tumors remain visible to immune surveillance.

The study of asparagine-related immune regulation is also conceptually valuable because it challenges the tendency to treat the amino acid landscape as fully mapped (181–184). Many current models focus heavily on a relatively small set of canonical metabolites, but the tumor microenvironment is metabolically diverse, and additional amino acid-dependent signaling circuits are likely to exist. Asparagine therefore serves as an example of how the field is evolving from a narrow catalog of well-known pathways toward a broader systems-level understanding of amino acid-mediated immune control (185, 186). From a translational perspective, emerging pathways such as asparagine signaling may prove especially valuable because they offer opportunities for innovation beyond heavily studied targets that have already encountered therapeutic limitations. At the same time, these newer axes require careful validation across tumor types and immune contexts. Their significance may vary according to tissue origin, metabolic background, and the composition of the tumor microenvironment. Even so, the recognition of asparagine as a potential immunoregulatory metabolite supports the broader argument of this review: amino acid metabolism should be understood as a dynamic network of signaling pathways that continues to expand in scope and therapeutic relevance.

**Figure 3:**
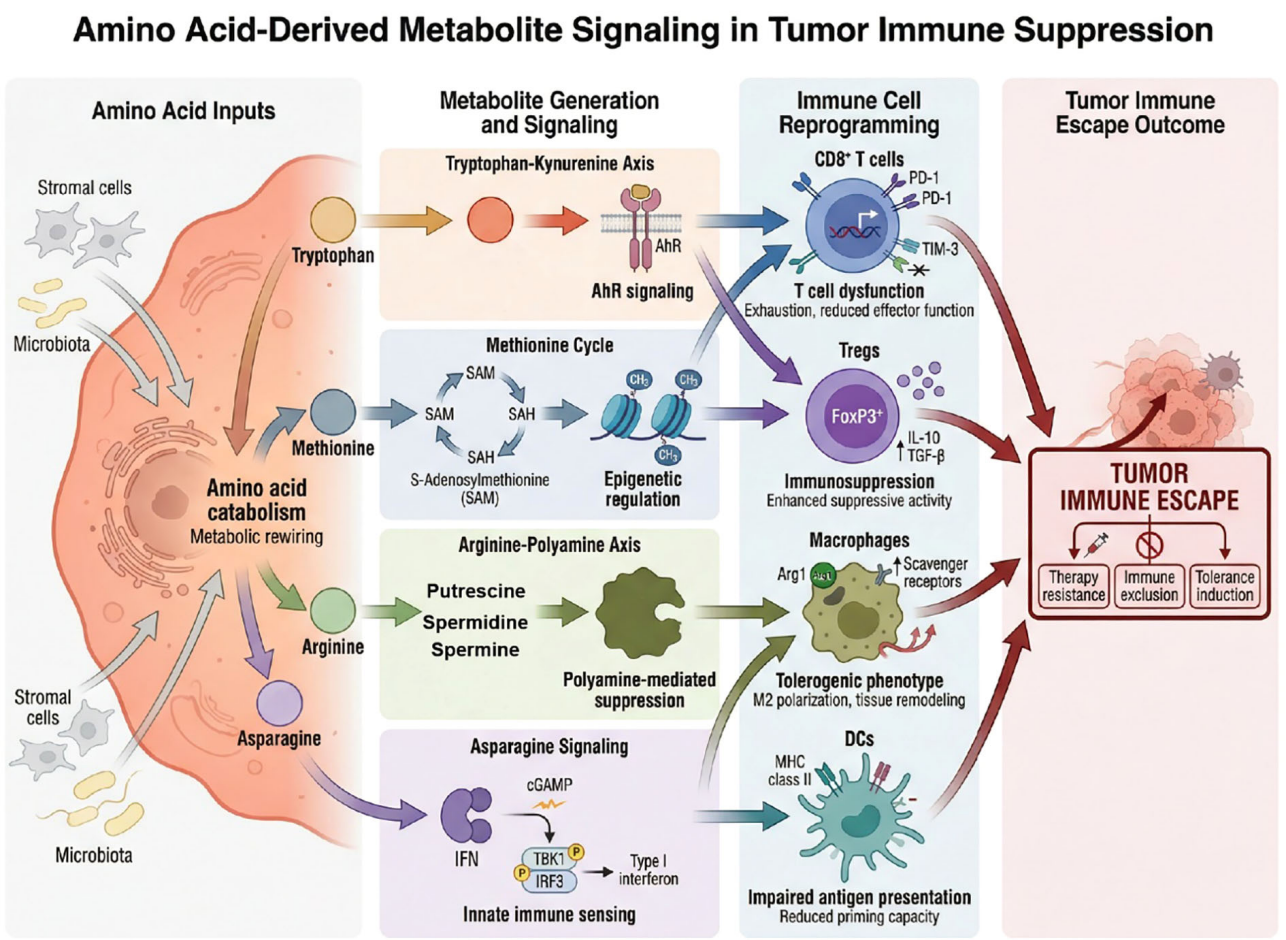
Amino acid-derived metabolite signaling in tumor immune suppression. Tumor-associated amino acid metabolism generates bioactive metabolites, including kynurenine, methyl donor-related intermediates, polyamines, and emerging asparagine-linked signals, which reshape immune-cell behavior. These metabolite-driven pathways promote T-cell dysfunction, suppressive myeloid polarization, impaired innate immune sensing, and immune tolerance, thereby reinforcing tumor immune escape.

Taken together, amino acid-derived metabolite signaling represents the second major layer of tumor immune escape. If nutrient competition defines who gains access to metabolic resources, metabolite signaling determines how those resources are interpreted and converted into biological instruction. Through pathways such as kynurenine–AhR activation, methionine cycle-dependent methylation control, polyamine-supported myeloid suppression, and emerging asparagine-linked immune signaling, tumors transform metabolic activity into coordinated suppressive communication. The next step in this framework is to examine how these signals, together with nutrient restriction, reshape immune cell identity and function at the level of durable cell-state rewiring. **Table 1** summarizes the major amino acid metabolic axes involved in tumor immune escape, highlighting their key regulators, cellular compartments, immunological consequences, and therapeutic relevance. **Figure 3** illustrates how amino acid catabolism generates bioactive metabolites that function as active immunoregulatory signals in the tumor microenvironment. Through kynurenine–AhR activation, methylation-dependent regulation, polyamine-mediated myeloid suppression, and emerging asparagine-linked signaling, tumors convert metabolic activity into coordinated immune suppression.

**Table 1:** Major amino acid metabolic pathways and regulatory mechanisms involved in tumor immune escape.

Amino acid axis	Key enzymes/transporters	Major cellular compartments involved	Main immunoregulatory mechanism	Immune consequence	Therapeutic implication	Representative PMID(s)
Tryptophan–kynurenine	IDO1, IDO2, TDO, AhR	Tumor cells, TAMs, DCs, T cells, microbiota-related inputs	Tryptophan depletion and kynurenine accumulation activate AhR-dependent tolerogenic programs	CD8+ T-cell dysfunction, Treg support, myeloid suppression, impaired antitumor immunity	IDO/TDO inhibition, AhR-targeted strategies, combination with immune checkpoint blockade	35121943; 35139353
Glutamine metabolism	SLC1A5, GLS, glutamine antagonists-related targets	Tumor cells, CD8+ T cells, MDSCs, TAMs	Tumor glutamine consumption restricts nutrient availability and supports anabolic and suppressive programs	Reduced T-cell proliferation and cytokine production; enhanced tumor growth and myeloid suppression	Glutamine antagonists, tumor-selective glutamine utilization blockade, combination immunotherapy	33320840; 35861366; 35930753; 35189139; 38536921
Methionine/one-carbon metabolism	Methionine transport systems, MAT-related metabolism, one-carbon metabolic enzymes, methylation machinery	Tumor cells, T cells, innate immune cells	Competition for methionine and altered methyl donor availability reshape epigenetic and transcriptional programs	T-cell exhaustion, impaired effector differentiation, altered innate immune sensing, context-dependent immune suppression	Methionine restriction/modulation, one-carbon metabolism targeting, epigenetic–immunometabolic combination strategies	33674593; 37267951; 37537369; 39672819; 39952933
Arginine metabolism	ARG1, iNOS, arginine transporters, polyamine synthesis enzymes	Tumor cells, TAMs, MDSCs, T cells	Arginine depletion limits T-cell fitness, while downstream metabolites such as polyamines reinforce suppressive myeloid programs	Impaired immune surveillance, reduced T-cell activation, macrophage-mediated immune suppression	ARG1 inhibition, arginine restoration strategies, polyamine pathway targeting	38942027; 39880485; 40185095
Asparagine metabolism	ASNS and related regulatory pathways	Tumor cells, innate immune signaling networks, T cells	Asparagine availability intersects with immune sensing and interferon-related pathways	Reduced immune visibility, impaired innate immune activation, support of tumor immune escape	Asparagine-targeted metabolic intervention, biomarker development for emerging immunometabolic axes	39964752
Microbiota-associated amino acid signaling	Microbial tryptophan-metabolizing enzymes and related pathways	Microbiota, TAMs, epithelial/stromal interfaces, immune cells	Microbiota-derived amino acid metabolites modulate host immune receptors and myeloid polarization	Reinforced immune tolerance, altered inflammatory tone, context-dependent immune suppression	Microbiota-informed immunometabolic therapy, combined metabolic and ecological targeting	35139353; 38942027; 37537369

## 5 Immune cell rewiring by reprogrammed amino acid metabolism

### 5.1 CD8+ T-cell dysfunction and exhaustion

Among the immune populations affected by amino acid metabolic reprogramming, CD8+ T cells are arguably the most consequential because they serve as the principal executors of direct antitumor immunity. Their ability to recognize malignant cells, expand clonally, produce effector cytokines, and mediate cytotoxic killing depends on a tightly coordinated metabolic program that must be sustained in the face of persistent antigen exposure (118, 187). Within tumors, however, this program is continuously challenged by nutrient deprivation, suppressive metabolites, hypoxia, and chronic inflammatory stress. Reprogrammed amino acid metabolism therefore acts as a central force driving CD8+ T-cell dysfunction. Glutamine deprivation provides a representative mechanism linking nutrient stress to mitochondrial dysfunction in CD8+ T cells. Insufficient glutamine limits glutamate and α-ketoglutarate production, thereby weakening TCA-cycle replenishment and mitochondrial respiration (188, 189). Under persistent tumor-associated glutamine restriction, CD8+ T cells may develop reduced mitochondrial membrane potential, impaired respiratory capacity, increased oxidative stress, and enhanced apoptosis. These mitochondrial defects translate into altered T-cell function, including reduced clonal expansion, decreased IL-2 and IFN-γ secretion, impaired granzyme/perforin-associated cytotoxicity, and weakened persistence within tumors. Therefore, glutamine insufficiency should be considered a direct metabolic driver of CD8+ T-cell dysfunction rather than merely a background feature of nutrient competition.

The effects of amino acid restriction on CD8+ T cells are multifaceted. Insufficient availability of key amino acids disrupts biosynthetic readiness, weakens mTOR-associated activation pathways, impairs mitochondrial adaptation, and reduces the production of effector molecules such as interferon-γ and granzyme-associated cytotoxic mediators (190, 191). Under acute conditions, these changes may manifest as incomplete activation or reduced proliferative capacity. Under chronic conditions, however, persistent metabolic stress contributes to the emergence of a more profound dysfunctional state characterized by diminished responsiveness, limited self-renewal, and progressive loss of effector potential. In this context, amino acid insufficiency is not merely inhibitory; it is instructive, helping to shape the developmental trajectory of T cells within tumors. This is particularly relevant to T-cell exhaustion (192–195). Exhaustion is often described primarily as a consequence of chronic antigen stimulation and persistent inhibitory receptor signaling, but metabolic conditions are increasingly recognized as crucial modifiers of this process. Reprogrammed amino acid metabolism can reinforce exhaustion by depriving T cells of the resources required to maintain effector identity while simultaneously exposing them to metabolites that stabilize dysfunctional transcriptional programs. Thus, exhaustion should be understood not only as a signaling-defined state but also as a metabolically conditioned fate. Amino acid deprivation and metabolite-mediated suppression together lower the threshold for exhaustion and reduce the likelihood that exhausted-like T cells can be rescued by checkpoint blockade alone. Another important consideration is that amino acid-mediated dysfunction may not affect all CD8+ T cells equally (196, 197). Distinct T-cell subsets within tumors differ in proliferative status, localization, and metabolic flexibility. Progenitor-like exhausted T cells, terminally exhausted cells, tissue-resident populations, and recently recruited effectors may each display different vulnerabilities to amino acid scarcity and metabolite exposure. This heterogeneity has direct therapeutic implications because some subsets may be more amenable to metabolic rescue than others. As a result, understanding CD8+ T-cell dysfunction in tumors requires attention not only to the presence of amino acid competition, but also to the timing, localization, and differentiation state in which that competition is experienced.

### 5.2 Regulatory T-cell metabolic adaptation and suppressive privilege

Regulatory T cells are particularly important in amino acid-mediated immune escape because they often retain suppressive activity under metabolic conditions that impair conventional effector T cells (198, 199). In nutrient-poor, hypoxic, and chronically inflamed tumor microenvironments, amino acid restriction does not suppress all lymphocyte subsets equally. Effector CD8+ T cells require strong anabolic metabolism to sustain proliferation, cytokine production, and cytotoxicity, whereas Tregs can maintain suppressive function through more flexible metabolic programs (200, 201). This differential adaptability creates a form of metabolic privilege, allowing Tregs to persist and function when tumoricidal lymphocytes become metabolically exhausted.

Several amino acid-related mechanisms contribute to this advantage. First, tryptophan catabolism supports Treg accumulation and stability through the kynurenine–AhR axis. IDO-expressing tumor cells, macrophages, MDSCs, and tolerogenic dendritic cells deplete tryptophan while producing kynurenine and related metabolites (202). These metabolites can activate AhR-dependent transcriptional programs that favor immune tolerance, promote Treg stability, and suppress effector T-cell expansion. Thus, the tryptophan–kynurenine pathway not only inhibits antitumor effector immunity but also actively reshapes the lymphocyte balance toward regulatory dominance.

Second, amino acid sensing pathways influence Treg fitness and functional programming. Arginine, leucine, and glutamine can modulate mTOR-related signaling, mitochondrial activity, redox balance, and biosynthetic capacity in T cells. Compared with highly proliferative effector T cells, Tregs may better tolerate fluctuations in amino acid availability and preserve suppressive function through adaptive metabolic rewiring (8, 44). However, this does not mean that Tregs are metabolically inactive. Instead, their persistence depends on selective use of nutrient-sensing pathways that support survival, FOXP3 expression, and inhibitory mediator production under stress conditions.

Methionine metabolism has also been proposed to influence Treg stability through methylation-dependent mechanisms, although direct evidence in tumor-infiltrating Tregs remains limited. Methionine-derived S-adenosylmethionine provides methyl donors for DNA, RNA, protein, and histone methylation. By controlling methylation potential, the methionine cycle may influence FOXP3-associated transcriptional stability and the maintenance of suppressive Treg programs (203, 204). In tumors with altered methionine availability, changes in methyl donor balance may therefore affect not only CD8+ T-cell exhaustion but also the persistence and stability of immunosuppressive Tregs.

These adaptive features have important therapeutic implications. Broad amino acid-targeted interventions may unintentionally preserve or enhance Treg dominance if they impair effector T cells more strongly than regulatory T cells. Conversely, strategies that selectively disrupt Treg-favorable metabolic pathways, such as IDO–kynurenine–AhR signaling or context-specific amino acid sensing, may help restore the balance between effector immunity and immune tolerance. Therefore, Treg metabolic adaptation should be considered a central component of amino acid-mediated immune escape and a key determinant of response to immunometabolic therapy.

### 5.3 Tumor-associated macrophages as amino acid signal interpreters

Tumor-associated macrophages occupy a central position in the amino acid metabolic network because they are not only responsive to metabolic conditions but also active interpreters and amplifiers of amino acid-derived signals (205, 206). Unlike effector T cells, which are often viewed primarily as victims of nutrient competition, macrophages function as adaptive sensors that translate metabolic cues into stable phenotypic programs. In tumors, this capacity frequently promotes immune suppression, tissue remodeling, angiogenesis, and support of malignant progression.

Amino acid-derived metabolites are especially important in shaping macrophage behavior. Signals generated from tryptophan catabolism, arginine turnover, and related metabolic pathways can influence transcriptional programs associated with tolerogenic and repair-oriented states. These macrophage phenotypes are often characterized by diminished proinflammatory function, enhanced capacity to suppress lymphocyte activation, and increased support for extracellular matrix restructuring and tumor invasion (207–210). Through these changes, macrophages become not merely passive components of the tumor microenvironment but active executors of amino acid-mediated immune escape. This role is particularly important because macrophages can reinforce the metabolic architecture that shaped them in the first place (211, 212). Once polarized into suppressive states, they may further consume nutrients, secrete inhibitory mediators, support abnormal vascular structure, and coordinate with fibroblasts or other myeloid cells to maintain a hostile environment for effector lymphocytes. Thus, amino acid metabolic signaling and macrophage polarization form a feed-forward loop: metabolites promote suppressive macrophage states, and suppressive macrophages further stabilize metabolically restrictive conditions. This loop helps explain why immune dysfunction in tumors is often persistent and spatially organized. Viewing tumor-associated macrophages as amino acid signal interpreters also clarifies why myeloid-targeted therapy may be necessary in addition to T-cell-directed approaches. Even if lymphocyte function can be partially restored, persistent suppressive macrophage states may continue to distort nutrient distribution and inhibitory signaling. Therefore, effective reversal of tumor immune escape may require disrupting the macrophage-centered metabolic circuits that convert amino acid-derived signals into long-lasting suppressive architecture.

### 5.4 Myeloid cell immunometabolism: IDO activity and glutamine utilization

Myeloid cells are central executors of amino acid-mediated immune suppression because they actively consume amino acids, generate immunoregulatory metabolites, and translate metabolic stress into stable suppressive phenotypes (67, 213). Among these mechanisms, IDO-dependent tryptophan catabolism is particularly important. In the tumor microenvironment, IDO activity is not restricted to malignant cells; tumor-associated macrophages, myeloid-derived suppressor cells, and tolerogenic dendritic cells can also express IDO in response to inflammatory cytokines, hypoxia, and tumor-derived signals (214, 215). Through the conversion of tryptophan into kynurenine and related metabolites, IDO-expressing myeloid cells simultaneously reduce local tryptophan availability and activate kynurenine–AhR signaling. This dual effect suppresses effector T-cell proliferation and cytokine production, favors Treg stability, and promotes tolerogenic myeloid polarization. Therefore, IDO should be viewed not only as a tumor-intrinsic metabolic enzyme but also as a myeloid-centered immunometabolic checkpoint that coordinates nutrient depletion, metabolite signaling, and immune tolerance.

Glutamine utilization represents another important layer of myeloid immunometabolic control. Although glutamine is often discussed in relation to tumor cell anabolism and T-cell activation, suppressive myeloid populations also rely on glutamine metabolism to maintain their survival, differentiation, and immunoregulatory activity (31, 90). Glutamine uptake and glutaminolysis can support mitochondrial metabolism, redox balance, nucleotide synthesis, and epigenetic remodeling in tumor-associated macrophages and MDSCs. In this context, glutamine metabolism helps sustain myeloid suppressive programs characterized by reduced antigen-presenting capacity, increased inhibitory mediator production, and enhanced suppression of effector lymphocytes (45, 119). Conversely, pharmacological or dietary modulation of glutamine metabolism may reprogram suppressive myeloid cells, reduce their inhibitory function, and improve responsiveness to immune checkpoint blockade. However, because glutamine is also required for activated T cells and dendritic-cell function, therapeutic targeting of this axis requires careful attention to cell type, timing, dose, and tumor context.

Taken together, IDO activity and glutamine utilization illustrate how myeloid cells actively shape the amino acid metabolic landscape of tumors. Rather than serving only as passive responders to nutrient deprivation, myeloid cells function as metabolic organizers that enforce immune suppression through amino acid consumption, metabolite production, and feedback stabilization of tolerogenic cell states. This myeloid-centered perspective is essential for understanding why amino acid-targeted therapies may need to be combined with myeloid reprogramming strategies or checkpoint blockade to achieve durable antitumor immunity.

### 5.5 MDSCs and dendritic cell dysfunction

Myeloid-derived suppressor cells and dendritic cells represent two additional immune compartments that are profoundly influenced by reprogrammed amino acid metabolism, although in different ways. MDSCs are specialized suppressor populations that actively exploit metabolic pathways to inhibit T-cell responses, whereas dendritic cells are essential initiators of adaptive immunity whose function can be undermined by nutrient scarcity and metabolite-mediated stress (216–221). Together, these populations illustrate how amino acid metabolic rewiring affects both the suppression of established immune responses and the failure to generate effective new ones. MDSCs are particularly relevant because they directly participate in the control of amino acid availability. Through high metabolic activity and enzymatic processing of key amino acids, they can intensify nutrient deprivation within tumors while producing suppressive molecular cues that weaken T-cell activation. This places MDSCs at the intersection of nutrient competition and metabolite signaling. Their function is not simply to respond to a suppressive environment, but to help enforce it. As these cells accumulate, they amplify amino acid restriction, reinforce inhibitory myeloid circuits, and contribute to the emergence of a tumor microenvironment in which effector immunity becomes progressively unsustainable.

Dendritic cells, by contrast, are metabolically sensitive antigen-presenting cells whose function depends on adequate support for antigen uptake, processing, migration, and T-cell priming. In amino acid-restricted tumors, dendritic cells may lose the metabolic stability needed to maintain these activities (222, 223). Suppressive metabolites can further compromise their maturation state or reduce their ability to provide co-stimulatory signals. The result is a failure of productive immune priming even when tumor antigens are present. In this way, reprogrammed amino acid metabolism affects not only the effector phase of immunity, but also its upstream initiation. The simultaneous expansion of MDSC-mediated suppression and impairment of dendritic cell function is especially damaging because it creates a dual deficit in cancer immunity. Existing effector responses are weakened, while new responses are less effectively generated. This coordinated disruption contributes substantially to immunotherapy resistance, particularly in tumors with dominant myeloid signatures. Therefore, amino acid metabolic rewiring should be recognized as a mechanism that destabilizes the entire immune response continuum, from antigen presentation to terminal cytotoxic function.

### 5.6 Metabolic symbiosis and immunosuppressive ecosystem formation

The effects of amino acid metabolic rewiring on immune cells are best understood not as isolated events occurring in separate cell types, but as components of a broader immunosuppressive ecosystem. Tumors do not escape immunity through a single metabolic lesion (43, 224). Instead, they build a coordinated network in which malignant cells, macrophages, MDSCs, Tregs, fibroblasts, dendritic cells, endothelial structures, and sometimes microbiota-related signals cooperate to produce a metabolically selective environment. Within this environment, nutrient access, metabolite exposure, and cell-state adaptation are interdependent. We propose that these partially characterized interactions can be interpreted as a form of metabolic symbiosis, although complete multicellular amino acid exchange networks have rarely been reconstructed experimentally in intact tumors.

Metabolic symbiosis does not imply equal benefit for all participating cells. Rather, it refers to a structured form of cooperation in which the metabolic activities of one cell population create conditions that support the suppressive or survival advantages of another. Tumor cells may consume nutrients aggressively, producing stress conditions that disable effector T cells. Macrophages and MDSCs may interpret or amplify these conditions, stabilizing suppressive myeloid states (225, 226). Tregs may persist under the same constraints and further dampen immunity. Stromal cells may shape spatial nutrient gradients and metabolite exchange, while vascular abnormalities prevent efficient resource redistribution. The result is an ecosystem in which immune suppression is maintained collectively rather than by any single cell type alone. This ecosystem model is important because it explains why reversing one element of immune dysfunction is often insufficient. Restoring nutrient access to T cells may not fully recover antitumor immunity if suppressive myeloid cells and Tregs remain dominant. Likewise, blocking a suppressive metabolite pathway may have limited benefit if tumor cells continue to monopolize amino acids and prevent dendritic cell priming. Available studies support several individual reinforcing interactions, but the extent of redundancy and compensation across the complete network remains incompletely resolved. In practical terms, this means that amino acid-targeted interventions are most likely to succeed when they account for the networked nature of tumor immunometabolism.

**Figure 4:**
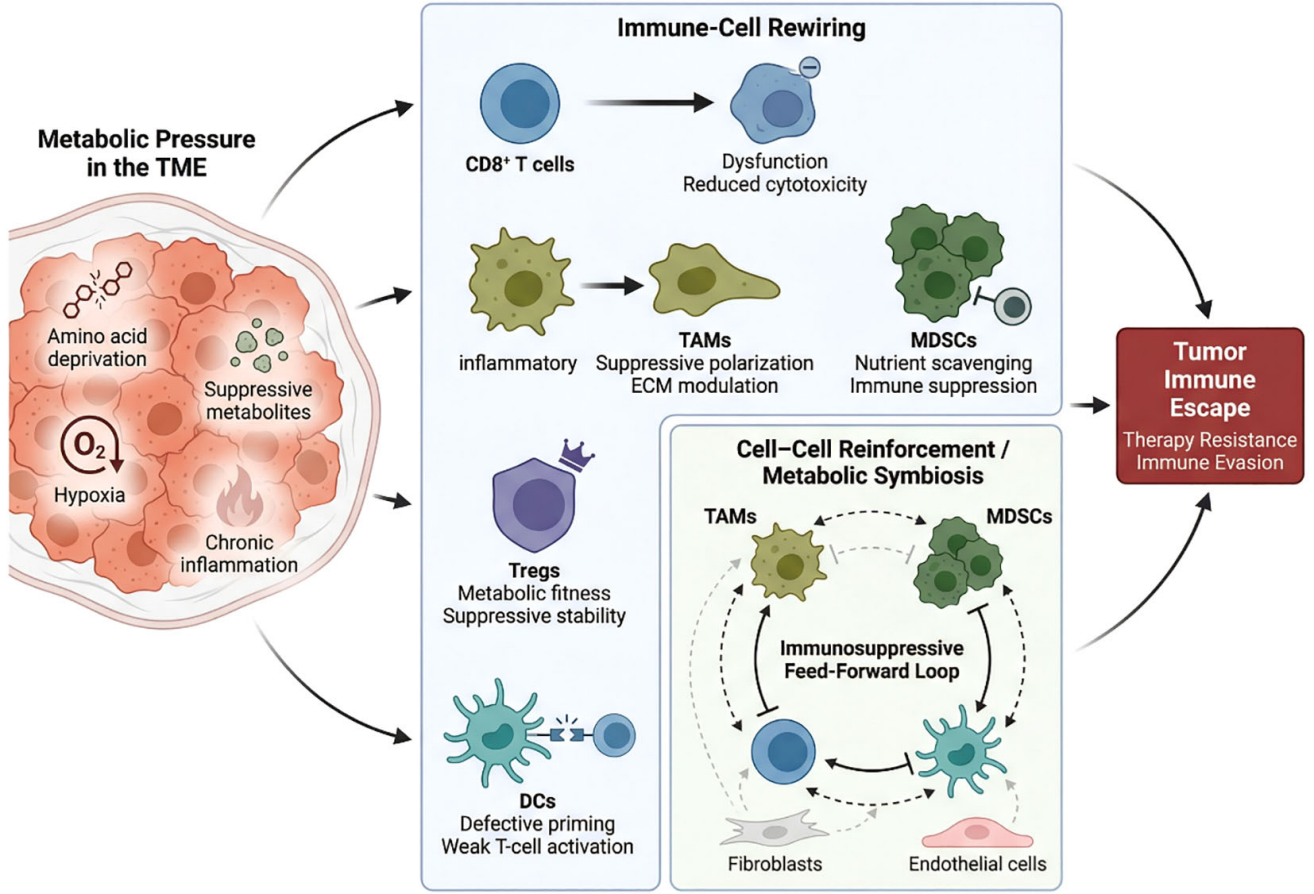
Immune cell rewiring by reprogrammed amino acid metabolism. Reprogrammed amino acid metabolism imposes nutrient deprivation, suppressive metabolite exposure, and chronic metabolic stress on the tumor microenvironment, thereby differentially reshaping immune-cell states. These changes promote CD8+ T-cell dysfunction, Treg persistence, suppressive myeloid polarization, dendritic-cell impairment, and metabolic symbiosis, ultimately driving tumor immune escape.

Conceptually, immunosuppressive ecosystem formation represents the culmination of the first three layers described in this review. Nutrient competition determines resource access, metabolite signaling defines how metabolic states are communicated, and immune cell rewiring stabilizes the resulting phenotypes. Together, these processes generate a metabolically governed immune landscape that sustains tumor persistence. This landscape also provides the mechanistic basis for therapeutic resistance, particularly resistance to interventions that rely on the functional restoration of immune effector cells. The next section therefore examines how amino acid metabolic rewiring contributes directly to resistance against immunotherapy. **Table 2** outlines how reprogrammed amino acid metabolism differentially reshapes lymphoid and myeloid compartments, ultimately favoring immune dysfunction, tolerance, and tumor persistence. **Figure 4** illustrates how reprogrammed amino acid metabolism differentially reshapes lymphoid and myeloid compartments in the tumor microenvironment. These coordinated changes establish an immunosuppressive ecosystem characterized by T-cell dysfunction, myeloid suppression, and impaired immune priming. 

**Table 2:** Impact of reprogrammed amino acid metabolism on immune cell populations in the tumor microenvironment.

Immune cell type	Major amino acid-related influences	Main functional changes	Consequence for antitumor immunity	Representative PMID(s)
CD8+ T cells	Deprivation of glutamine, methionine, arginine, and tryptophan; exposure to kynurenine and other suppressive metabolites	Reduced proliferation, impaired cytokine production, weakened cytotoxicity, mitochondrial stress, exhaustion-like reprogramming	Loss of direct tumor-killing capacity and poor response to immune checkpoint blockade	33320840; 33674593; 35121943; 38536921; 40185095
CD4+ effector T cells	Amino acid insufficiency and suppressive metabolite signaling	Impaired activation and helper function, weakened support for CD8+ T-cell and DC responses	Reduced coordination of adaptive antitumor immunity	33674593; 35139353; 39880485
Regulatory T cells (Tregs)	Relative adaptation to nutrient-poor and suppressive environments; support from tryptophan-derived signaling	Preserved survival and suppressive function under metabolic stress	Increased immune tolerance and suppression of effector lymphocytes	35121943; 35139353
Tumor-associated macrophages (TAMs)	Kynurenine–AhR signaling, arginine-derived polyamines, broader amino acid-derived suppressive cues	Polarization toward tolerogenic, tissue-remodeling, tumor-supportive phenotypes	Strengthened immune suppression, matrix remodeling, angiogenesis, and support of tumor progression	35139353; 40185095; 39880485
Myeloid-derived suppressor cells (MDSCs)	Amino acid consumption and enzymatic processing, especially along arginine and glutamine-associated pathways	Reinforced suppressive activity, intensified nutrient depletion, production of inhibitory signals	Amplified T-cell suppression and sustained immunosuppressive microenvironment	35861366; 40185095
Dendritic cells (DCs)	Nutrient deprivation and suppressive metabolite exposure	Impaired antigen uptake, processing, maturation, migration, and co-stimulatory signaling	Defective T-cell priming and weakened initiation of antitumor immunity	35121943; 39964752
NK cells	Amino acid limitation and metabolically hostile tumor conditions	Reduced activation and cytotoxicity	Decreased innate immune surveillance against tumor cells	38536921; 39964752
Immune ecosystem as a whole	Multicellular nutrient competition and metabolite exchange among tumor, stromal, and immune cells	Stabilization of dysfunctional and suppressive cell states	Formation of a metabolically governed immunosuppressive ecosystem that supports tumor persistence	35139353; 37537369; 38942027; 40185095

### 5.7 Cross-pathway crosstalk and feedback regulation within amino acid metabolic networks

Although individual amino acid pathways are often discussed separately for clarity, they function in tumors as interconnected regulatory networks rather than isolated metabolic routes. Crosstalk can occur at several levels, including shared transport systems, competition for metabolic cofactors, convergent signaling nodes, epigenetic regulation, and immune-cell feedback loops (8, 44). Therefore, the immunological effect of one amino acid axis may depend on the activity of another pathway and on the cellular compartment in which these pathways are engaged.

One important form of crosstalk occurs through convergent control of T-cell fitness. Glutamine metabolism supports α-ketoglutarate production, TCA-cycle anaplerosis, mitochondrial respiration, redox balance, and mTORC1–c-Myc-dependent effector programming (31, 227). Methionine metabolism, in contrast, provides S-adenosylmethionine for methylation-dependent maintenance of effector transcriptional and chromatin states. These two pathways therefore regulate different but complementary dimensions of T-cell competence: glutamine supports bioenergetic and biosynthetic capacity, whereas methionine supports epigenetic fitness. When both glutamine and methionine are limited in nutrient-restricted tumor niches, CD8+ T cells may simultaneously lose mitochondrial function and effector-associated chromatin accessibility, thereby becoming more vulnerable to exhaustion and less responsive to checkpoint blockade.

A second network module involves myeloid-centered amino acid catabolism. Tryptophan catabolism through IDO/TDO generates kynurenine, which activates AhR-dependent tolerogenic programs, whereas arginine metabolism through ARG1 and downstream polyamine synthesis supports suppressive myeloid survival, tissue remodeling, and T-cell inhibition (228, 229). These pathways can reinforce one another because tolerogenic macrophages and MDSCs may simultaneously consume amino acids, produce inhibitory metabolites, and stabilize immune-suppressive transcriptional states. In this setting, tryptophan and arginine metabolism do not act as independent suppressive axes; rather, they cooperate to establish a myeloid-dominant metabolic niche that limits T-cell priming, expansion, and cytotoxic activity.

Feedback loops further strengthen this network. Amino acid-derived metabolites can polarize macrophages, MDSCs, dendritic cells, and Tregs toward suppressive states. Once established, these cells can further remodel the amino acid landscape by consuming nutrients, expressing catabolic enzymes, secreting inhibitory mediators, and altering stromal or vascular function (8, 230). For example, suppressive myeloid cells may reinforce tryptophan depletion, arginine consumption, and polyamine accumulation, while Tregs may preserve immune tolerance under conditions that impair effector T cells. Thus, amino acid metabolic regulation is self-amplifying: metabolic stress induces suppressive immune states, and suppressive immune states further stabilize metabolic stress.

Microenvironmental and tissue-level factors also connect amino acid pathways. Poor perfusion, dense extracellular matrix, hypoxia, endothelial dysfunction, and cancer-associated fibroblast activity can simultaneously restrict multiple amino acids and alter metabolite diffusion. In mucosal or gastrointestinal tumor settings, microbiota-derived metabolites may additionally interact with tryptophan–AhR signaling and myeloid activation (215, 231). These spatial and ecological factors help explain why amino acid metabolic networks are tumor-type and niche dependent rather than uniform across cancers. This network perspective has important therapeutic implications. Targeting a single amino acid pathway may be insufficient if parallel pathways compensate or if suppressive immune cells preserve the metabolic niche. For example, inhibition of IDO-driven tryptophan catabolism may have limited efficacy when arginine metabolism, polyamine production, or glutamine-dependent myeloid suppression remains dominant. Similarly, glutamine-targeted therapy may require careful integration with strategies that preserve effector T-cell metabolism while suppressing tumor and myeloid metabolic dominance (232, 233). Therefore, future therapeutic strategies should identify dominant network nodes, compensatory pathways, and feedback loops before selecting amino acid-targeted combinations.

## 6 Amino acid metabolism and immunotherapy resistance

### 6.1 Metabolic barriers to checkpoint blockade

Checkpoint blockade removes inhibitory receptor signals but does not restore the nutrients, mitochondrial capacity, or epigenetic plasticity required for immune-cell recovery. Consequently, amino acid deprivation and suppressive metabolite accumulation can maintain T-cell dysfunction even after PD-1/PD-L1 or CTLA-4 signaling is inhibited (234, 235). Amino acid metabolic dysregulation therefore constitutes a parallel barrier to effective immune reinvigoration.

The problem extends beyond CD8+ T cells alone. Dendritic cells must maintain sufficient metabolic competence to prime and restimulate antitumor lymphocytes, while macrophages and MDSCs can preserve suppressive programs that continue to dampen immunity during treatment (236, 237). If amino acid metabolic reprogramming remains uncorrected, checkpoint blockade may act on a microenvironment in which antigen presentation is suboptimal, myeloid suppression persists, and regulatory populations retain their dominance. In such cases, immunotherapy encounters a tissue ecosystem that is structurally resistant to immune reinvigoration. This perspective is especially important because it reframes immunotherapy resistance as a problem of functional context rather than receptor status alone. The question is not simply whether inhibitory pathways are present, but whether immune cells have the metabolic capacity to take advantage of their release. Amino acid metabolism therefore represents a foundational layer of resistance that can operate upstream, in parallel with, or downstream of checkpoint signaling. Understanding this layer is essential for interpreting why some tumors are primarily refractory to treatment, why others acquire resistance over time, and why combinatorial strategies that include metabolic intervention may be necessary to achieve meaningful clinical benefit.

### 6.2 Amino acid-related signatures of resistance

As the role of immunometabolism in therapy resistance becomes clearer, amino acid-related molecular and cellular signatures are increasingly relevant as indicators of treatment responsiveness. These signatures may include altered expression of amino acid transporters, activation of metabolic enzymes, accumulation of amino acid-derived metabolites, epigenetic features linked to one-carbon metabolism, or immune cell states shaped by chronic nutrient stress (238, 239). Together, they provide a framework for understanding how amino acid metabolic reprogramming contributes to heterogeneous responses to immunotherapy across tumor types and patient populations.

One important category of resistance signatures involves T-cell state transitions. Exhausted or dysfunctional T cells are not metabolically uniform; rather, they exist along a spectrum of differentiation states with distinct biosynthetic needs and rescue potential (240). Amino acid metabolic features, including those related to methionine-dependent methylation and one-carbon metabolism, may help define whether a T-cell compartment remains plastic enough to respond to checkpoint blockade or has progressed toward a more terminally fixed dysfunctional state. In this way, amino acid-associated programs are not merely correlates of immune dysfunction but may serve as mechanistic markers of reversibility versus irreversibility.

Another major category includes myeloid-centered signatures. Tumors enriched in suppressive macrophages or MDSCs often display metabolic profiles consistent with amino acid depletion, catabolite accumulation, and reinforced tolerogenic signaling. Such features may identify microenvironments in which checkpoint blockade alone is unlikely to overcome dominant suppressive circuits (211, 212). Similarly, the presence of tryptophan catabolism-associated programs, arginine-processing pathways, or polyamine-linked myeloid remodeling may signal an ecosystem-level resistance state in which immunotherapy is constrained by broader metabolic governance rather than a lack of immune recognition per se. Metabolite-based signatures may also prove informative. Ratios reflecting altered tryptophan metabolism, patterns of methyl donor-associated metabolites, and other amino acid-related biochemical states could potentially serve as integrated readouts of tumor–immune metabolic interaction. Although such biomarkers require further validation, they are attractive because they may capture both tumor-intrinsic and microenvironmental regulation at once. Ultimately, amino acid-related resistance signatures are valuable not only for predicting response but also for identifying which immunometabolic mechanisms should be prioritized in combination therapy.

### 6.3 Determinants of response heterogeneity across tumors

Not all tumors rely on the same amino acid metabolic strategies, and not all immune microenvironments respond to metabolic pressure in the same way. This variability is a major reason why the impact of amino acid metabolism on immunotherapy differs across tumor types, disease stages, and individual patients. Response heterogeneity reflects differences in tumor genetics, lineage-specific metabolic wiring, tissue nutrient composition, stromal architecture, vascular function, microbiota influences, and immune cell composition (241, 242). Amino acid metabolism must therefore be interpreted as a context-dependent determinant of immunotherapy outcome rather than a universal mechanism with identical consequences in every setting. Tumor-intrinsic properties are one source of heterogeneity. Distinct cancers vary in their dependence on glutamine, methionine, tryptophan, arginine, or asparagine pathways, and these preferences shape how strongly they compete with immune cells for particular substrates. Genetic alterations may further intensify or redirect metabolic flux, altering the degree to which tumors generate suppressive metabolites or impose nutrient deprivation. These differences mean that the same immunotherapy may encounter very different amino acid landscapes depending on tumor lineage and molecular subtype.

Tumor-type-specific differences are particularly important when interpreting amino acid immunometabolism. For example, tumors arising in barrier tissues or gastrointestinal organs may be more strongly influenced by microbiota-associated tryptophan metabolites and local inflammatory tone (243, 244). Liver tumors develop within a metabolically specialized organ that strongly regulates amino acid, urea-cycle, and one-carbon metabolism, making systemic nutrient flux and hepatic immune tolerance especially relevant (245, 246). Pancreatic and other desmoplastic tumors may impose amino acid restriction through dense stromal architecture, poor perfusion, and fibroblast-mediated metabolic compartmentalization (247–249). Brain tumors are shaped by the blood–brain barrier, specialized nutrient transport, and distinct myeloid populations, which may alter how amino acid availability affects immune infiltration and activation (250, 251). These examples illustrate that amino acid metabolic regulation should be interpreted according to tissue origin, metabolic architecture, stromal organization, and immune context rather than treated as a uniform process across cancers.

The surrounding tissue context is equally important. Tumors arising in different organs are embedded in distinct physiological nutrient environments and interact with unique stromal and immune architectures. The liver, lung, gastrointestinal tract, brain, and omentum, for example, each present different baseline metabolic and immunological conditions that influence how amino acid competition is organized and how immune cells adapt (252–254). Spatial heterogeneity within the same tumor adds another layer of complexity, as regions with poor perfusion, dense fibrosis, or dominant myeloid infiltration may be metabolically and immunologically distinct from more accessible or inflamed zones. Immune composition also shapes response heterogeneity. A tumor enriched in progenitor-like exhausted T cells may be more amenable to metabolic rescue than one dominated by terminally dysfunctional T cells. Likewise, a microenvironment driven mainly by suppressive myeloid circuits may require different metabolic interventions from one in which nutrient competition between tumor cells and lymphocytes is the dominant problem. These considerations emphasize that amino acid metabolism should not be viewed as a single resistance factor, but as a contextual organizer of different resistance patterns.

**Figure 5:**
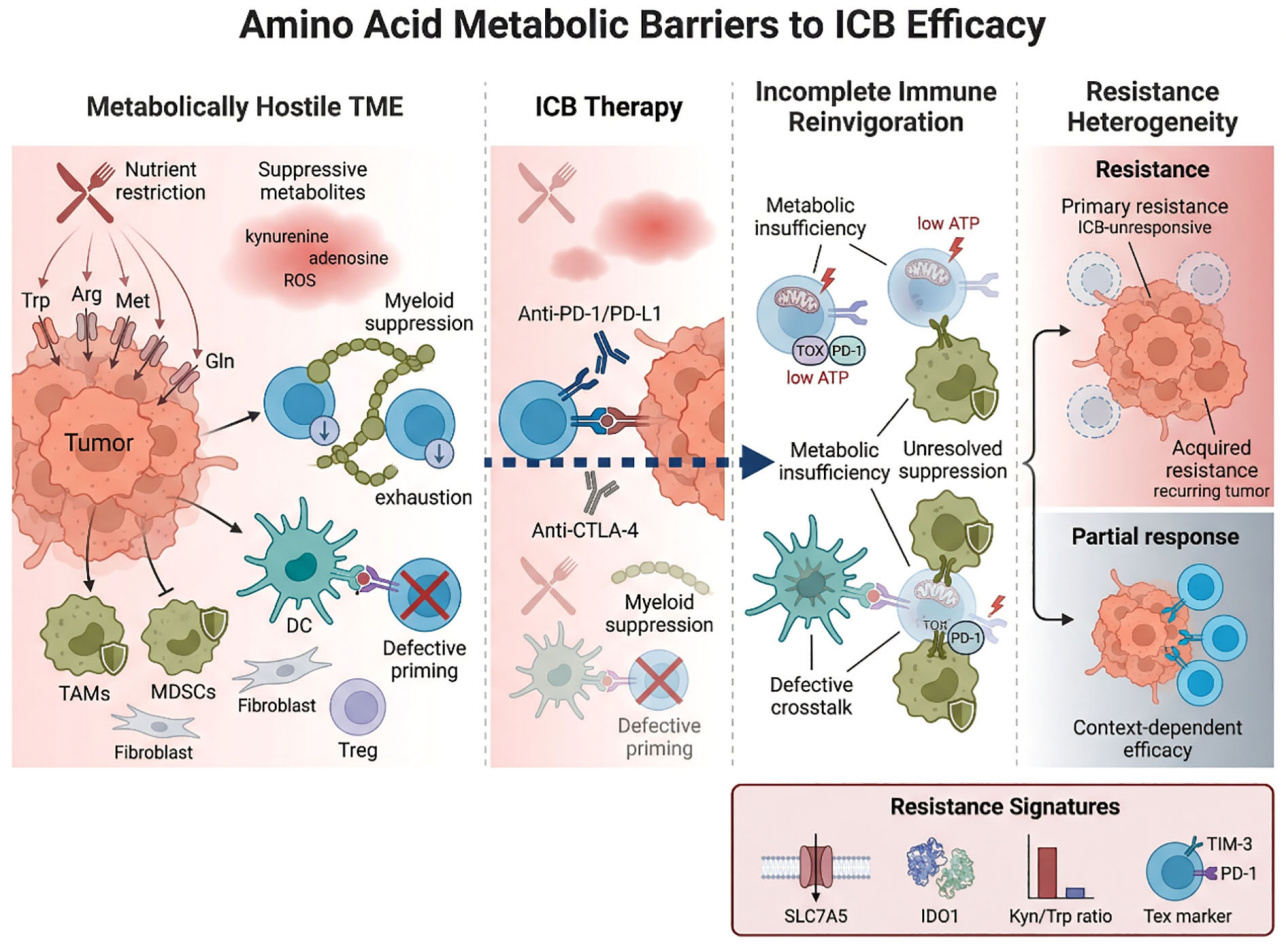
Amino acid metabolism as a barrier to immunotherapy. Amino acid deprivation, suppressive metabolites, defective antigen presentation, and persistent myeloid suppression create a metabolically hostile tumor microenvironment in which checkpoint blockade cannot fully restore antitumor immunity. This context leads to incomplete immune reinvigoration, persistent T-cell dysfunction, and heterogeneous responses ranging from partial benefit to primary or acquired resistance.

Taken together, heterogeneity in amino acid metabolism helps explain why some patients benefit from checkpoint blockade, why others require combination approaches, and why broad metabolic interventions may succeed in one cancer setting but fail in another. A more precise understanding of these differences will be essential for translating immunometabolic insights into clinically effective, patient-specific strategies. **Figure 5** illustrates how amino acid metabolic dysregulation limits the efficacy of immune checkpoint blockade by maintaining immune cells in a metabolically compromised state. Even when inhibitory receptor signaling is relieved, incomplete immune reinvigoration may still result in persistent dysfunction and therapy resistance.

## 7 Therapeutic targeting of amino acid metabolic networks

Although amino acid metabolism provides multiple therapeutically actionable nodes, mechanistic tractability should not be equated with clinical feasibility. The translational value of a given target depends on whether the pathway is dominant in the selected tumor, whether compensatory metabolic routes remain available, and whether tumor cells can be inhibited without compromising metabolically dependent immune populations. Accordingly, the following sections evaluate both the therapeutic rationale and the principal biological constraints of each strategy.

### 7.1 Targeting transporters and tumor amino acid utilization

Because tumors often secure their metabolic advantage through enhanced nutrient acquisition, one logical therapeutic approach is to target amino acid transporters or tumor-specific pathways of amino acid utilization. This strategy aims to reduce tumor metabolic dominance, increase local nutrient accessibility, and weaken the biosynthetic or signaling programs that sustain immune escape. In principle, restricting tumor access to key amino acids could not only inhibit malignant growth directly but also rebalance the microenvironment in favor of immune effector function. The appeal of this approach lies in its upstream position within the immunometabolic network. By interfering with transporter expression or nutrient utilization before suppressive metabolites accumulate or immune dysfunction becomes deeply fixed, such interventions may disrupt the earliest stages of metabolic immune escape (41, 99, 255–257). In tumors highly dependent on glutamine, methionine, or other specific amino acids, this could reduce competition pressure on infiltrating lymphocytes and indirectly support their activation and persistence. The principal limitation of transporter-directed therapy is the absence of strict tumor specificity (17). Transporters involved in glutamine, leucine, methionine, and other amino acid uptake are frequently induced not only in malignant cells but also in activated T cells, dendritic cells, and other immune populations undergoing anabolic reprogramming (258, 259). Consequently, systemic transporter inhibition may reduce tumor nutrient acquisition while simultaneously impairing lymphocyte expansion, cytokine production, antigen presentation, or memory formation (92). The therapeutic window is further narrowed by transporter redundancy, because blockade of one uptake route may induce alternative transporters or increase *de novo* amino acid synthesis. Moreover, transporter dependence varies substantially across tumor types and may change after treatment (19, 260). Therefore, expression alone is insufficient to establish therapeutic vulnerability; functional flux measurements, cellular localization, and evidence of non-redundant pathway dependence are required. Tumor-restricted delivery, conditional prodrugs, or targeting of lineage-specific transporter dependencies may be more feasible than systemic pathway-wide inhibition (261).

### 7.2 Blocking immunosuppressive amino acid catabolism

A second major strategy is to inhibit the catabolic pathways that convert amino acids into suppressive metabolites or deplete them from the tumor microenvironment. This approach has attracted strong interest because pathways such as tryptophan catabolism, arginine processing, and downstream polyamine production provide a clear mechanistic bridge between metabolism and immune suppression (152, 262). Blocking these pathways offers the possibility of preserving nutrient availability while simultaneously preventing the generation of immunoregulatory signals that stabilize tolerance.

Inhibiting immunosuppressive amino acid catabolism is mechanistically appealing because it may simultaneously preserve the parent nutrient and reduce the generation of inhibitory metabolites. However, this dual rationale has not consistently translated into clinical benefit (155, 263). The tryptophan pathway illustrates the problem of metabolic redundancy: inhibition of IDO1 may be bypassed by TDO2 activity, alternative AhR ligands, microbial metabolites, or other immunosuppressive pathways that maintain tolerogenic signaling (153, 264). Similarly, inhibition of ARG1 may not fully restore arginine availability when ARG2 activity, tumor-cell uptake, stromal metabolism, or downstream polyamine production remains active (216, 265). The cellular source of the relevant enzyme also matters, because tumor-cell and myeloid-cell catabolism may have different spatial distributions and therapeutic sensitivities. These mechanisms are unlikely to be equally dominant across all tumor types; therefore, enzyme expression alone should not be used as evidence of pathway dependence. Clinical development will require pharmacodynamic confirmation of target inhibition, measurement of substrate and metabolite changes, and patient selection based on functional pathway activity rather than isolated biomarker expression.

### 7.3 Rewiring methionine and one-carbon metabolism

The methionine and one-carbon metabolic network presents a particularly interesting therapeutic opportunity because of its links to epigenetic control, innate immune activation, and long-term immune cell state stability. Intervening in this axis offers the possibility of shifting both tumor and immune programs through changes in methyl donor availability and methylation-dependent regulation. Rather than simply starving tumors of a nutrient, strategies targeting methionine metabolism may reconfigure how malignant cells and immune cells interpret and encode metabolic information (266, 267). This potential is therapeutically attractive because it opens routes to influence immune visibility and response quality at multiple levels. Rewiring methionine metabolism may alter tumor cell chromatin states, affect the expression of immunoregulatory molecules, or enhance innate immune sensing pathways that support antitumor immunity (268). At the same time, however, methionine restriction or disruption of sulfur-related metabolic balance can also impair immune function if applied in ways that compromise lymphocyte fitness or broader host metabolism. This makes the methionine axis fundamentally different from simpler nutrient-deprivation models: it is not a straightforward “less is better” target, but a context-dependent regulatory system that may require precise modulation rather than blunt inhibition. The translational significance of this pathway lies in its bidirectionality. Methionine-targeted strategies may be beneficial when they destabilize tumor-promoting epigenetic programs or improve immune recognition, yet harmful when they deepen immune cell insufficiency. Therefore, successful therapeutic use of this axis will likely depend on carefully chosen treatment windows, rational combinations, and biomarkers that reveal whether a given tumor ecosystem is more likely to respond with immune activation or immune compromise.

### 7.4 Glutamine antagonists as immunometabolic sensitizers

Among currently explored immunometabolic strategies, targeting glutamine metabolism has emerged as one of the most compelling because of glutamine’s central role in tumor biosynthesis, myeloid cell function, and T-cell activation. Glutamine antagonists and related interventions may act as immunometabolic sensitizers by weakening tumor growth programs while also making the microenvironment more permissive to antitumor immunity (269–271). In some settings, this approach can reduce tumor metabolic dominance, diminish suppressive myeloid activity, and enhance the effectiveness of checkpoint blockade (272). The concept of sensitization is important. Rather than expecting glutamine-targeted therapies to function solely as standalone antitumor agents, they may be better understood as interventions that recondition the tumor ecosystem so that other therapies, particularly immunotherapies, can work more effectively. By reducing the intensity of nutrient competition and altering suppressive metabolic circuits, glutamine antagonism may increase the fraction of immune cells capable of responding to reinvigoration signals. Nevertheless, glutamine targeting also exemplifies the balancing act of immunometabolic therapy. Because activated lymphocytes require glutamine, indiscriminate inhibition may blunt the very responses one hopes to rescue. The most promising applications are therefore likely to be those that exploit differential metabolic dependencies, partial rather than absolute pathway suppression, or combination regimens in which immune activation is supported while tumor and suppressive myeloid metabolism are selectively constrained. This makes glutamine antagonists especially relevant as part of rationally designed, network-aware therapeutic strategies.

### 7.5 Rational combination strategies with immunotherapy

Given the networked nature of amino acid metabolic immune escape, combination therapy is likely to be more effective than single-pathway intervention in many settings. The rationale is straightforward: checkpoint blockade can release inhibitory signaling, but without correcting the metabolic context, immune recovery may remain incomplete. Conversely, metabolic intervention may improve nutrient access or reduce suppressive signaling, but without adequate immune activation, these changes may not translate into durable tumor control. Combining amino acid-targeted strategies with immunotherapy therefore offers a way to address both immune signaling and immune fitness simultaneously (273–275). Several types of combinations are conceptually attractive. Metabolic inhibitors may be paired with PD-1/PD-L1 blockade to enhance the reinvigoration of metabolically constrained T cells. Interventions that alter methionine-related epigenetic control may be combined with therapies that rely on improved tumor immunogenicity or innate immune sensing. Strategies targeting suppressive myeloid metabolism may be combined with agents that expand or recruit cytotoxic lymphocytes. In addition, microbiota-informed or stromal-modifying approaches could theoretically be integrated where amino acid metabolism is strongly shaped by ecological context rather than tumor-intrinsic pathways alone. The main advantage of combination therapy is that it acknowledges the layered organization of tumor immune escape. Nutrient competition, metabolite signaling, and immune cell rewiring do not operate independently, so successful therapy may need to intervene at more than one level. Rational combinations should therefore be guided by the dominant mechanism within a given tumor ecosystem rather than applied in a one-size-fits-all manner.

### 7.6 Challenges and limitations in translation

The first barrier is the limited cellular selectivity of metabolic intervention. Amino acid uptake, glutaminolysis, one-carbon metabolism, and nutrient-sensing pathways are required not only by malignant cells but also by activated lymphocytes, dendritic cells, hematopoietic progenitors, and normal proliferating tissues (17, 258). The same inhibitor may therefore suppress tumor growth in one cellular compartment while weakening immune activation in another. This creates a narrow and dynamic therapeutic window that cannot be predicted from tumor-cell monoculture experiments alone. Cell-specific genetic models, immune-competent systems, and direct assessment of immune-cell metabolic fitness are necessary before systemic targeting can be considered clinically justified.

The second barrier is pathway redundancy. Tumors may respond to blockade of one transporter or enzyme by increasing alternative amino acid uptake, activating salvage or biosynthetic pathways, exchanging metabolites with stromal cells, or shifting their dependence toward glucose, lipids, or other amino acids (19, 32). Immune suppression is similarly redundant: inhibition of one catabolic pathway may leave checkpoint signaling, hypoxia, lactate accumulation, suppressive cytokines, or parallel myeloid mechanisms intact (276). Thus, biochemical target inhibition does not necessarily indicate disruption of the broader immunosuppressive network. Longitudinal metabolic profiling will be required to detect compensatory responses and to determine whether combination treatment is necessary.

The third barrier is tumor-type specificity. Amino acid dependence is shaped by tissue of origin, oncogenic genotype, vascular supply, metastatic site, stromal composition, microbiota exposure, and previous treatment (277, 278). A pathway that is dominant in one cancer subtype may be dispensable in another, and metabolic dependencies may differ between primary tumors and metastatic lesions or change during treatment. Patient selection should therefore rely on evidence of functional pathway dependence rather than histology or enzyme expression alone. Ideally, transcriptomic and protein markers should be integrated with metabolite ratios, isotope-tracing data, spatial localization, and treatment-induced pathway changes.

The fourth barrier concerns dose, timing, and drug distribution. Transient or partial inhibition may reduce tumor metabolic fitness while preserving immune function, whereas prolonged systemic deprivation may suppress immune priming, effector expansion, and tissue homeostasis (25, 261). The effect may also differ when treatment is administered before checkpoint blockade, during T-cell expansion, or after resistant immune states have become established. Tumor-restricted delivery, conditional prodrugs, intermittent dosing, and pharmacodynamic monitoring may help separate antitumor effects from immune toxicity. These considerations indicate that amino acid-directed therapy should be developed as a precision intervention matched to a defined tumor ecosystem, rather than as uniform metabolic deprivation across unselected patients. **Table 3** summarizes current and emerging therapeutic strategies targeting amino acid metabolic networks, with emphasis on their immunometabolic rationale, translational potential, and major clinical challenges.

**Table 3:** Current and emerging therapeutic strategies targeting amino acid metabolic networks in cancer immunotherapy.

Therapeutic strategy	Representative targets/approaches	Intended immunometabolic effect	Potential advantage	Major limitation/challenge	Representative PMID(s)
Inhibition of tryptophan catabolism	IDO1/TDO inhibition, modulation of kynurenine–AhR signaling	Reduce suppressive metabolite signaling and relieve tryptophan-related immune inhibition	May restore immune responsiveness and improve checkpoint blockade efficacy	Pathway redundancy, variable clinical benefit, patient-selection issues	35121943; 35139353
Targeting glutamine metabolism	GLS inhibition, glutamine antagonists, blockade of tumor glutamine utilization	Reduce tumor metabolic dominance, weaken suppressive myeloid programs, improve T-cell function	Strong rationale for combination with immunotherapy	Shared glutamine dependence between tumor and immune cells may narrow therapeutic window	33320840; 35861366; 35930753; 35189139; 38536921
Modulating methionine/one-carbon metabolism	Dietary methionine modulation, one-carbon pathway targeting, methylation-related intervention	Reprogram tumor epigenetic states and immune signaling, potentially enhance tumor immunogenicity	Connects metabolism with epigenetic and innate immune regulation	Context-dependent effects; immune cells may also be harmed by excessive restriction	37267951; 37537369; 39672819; 39952933
Restoring arginine availability or blocking suppressive arginine metabolism	ARG1 inhibition, arginine supplementation/repletion strategies, targeting arginine-related suppressive pathways	Improve T-cell activation and reduce myeloid-mediated immune suppression	May enhance immune surveillance and T-cell fitness	Complex multicellular arginine turnover and possible compensatory pathways	39880485; 40185095; 38942027
Polyamine pathway targeting	Inhibition of polyamine synthesis or related downstream pathways	Weaken macrophage- and myeloid-associated suppressive phenotypes	Useful for disrupting myeloid-centered immune escape circuits	Limited specificity and incomplete understanding of context-dependent effects	40185095
Transporter-targeted intervention	Inhibition of amino acid transport systems preferentially used by tumors	Limit tumor nutrient acquisition and reduce competitive pressure on immune cells	Acts upstream in the metabolic network	Transporters may be shared by activated immune cells, risking collateral immune suppression	33320840; 35930753
Combination with immune checkpoint blockade	Amino acid-targeted therapy plus anti-PD-1/PD-L1 or anti-CTLA-4	Simultaneously improve immune fitness and relieve inhibitory signaling	Most rational strategy for metabolically resistant tumors	Requires biomarker-guided selection and precise treatment scheduling	38536921; 39672819; 39964752
Microbiota-informed immunometabolic intervention	Modulation of microbiota-derived amino acid metabolism or metabolite signaling	Reshape host–tumor metabolic communication and suppressive immune tone	Expands therapy beyond tumor-intrinsic metabolism	Mechanistic complexity, ecological variability, and translational uncertainty	35139353; 37537369; 38942027
Biomarker-guided precision immunometabolic therapy	Use of metabolic enzymes, transporters, metabolites, and multi-omics profiling	Match therapy to the dominant amino acid metabolic architecture of a given tumor	Improves precision and may reduce ineffective treatment	Biomarkers remain under development and require validation across tumor types	39952933; 39964752; 40185095

## 8 Biomarkers and translational opportunities

### 8.1 Enzymes and transporters as biomarkers

Amino acid metabolic enzymes and transporters are attractive biomarker candidates because they sit at key control points linking nutrient availability, suppressive metabolite production, and cellular metabolic dominance. Their expression levels may provide information about which pathways are active within tumors and which metabolic mechanisms are most likely to influence immune behavior. In principle, high expression of amino acid transporters or catabolic enzymes could identify tumors that strongly depend on particular amino acid axes and may therefore be vulnerable to pathway-targeted intervention (279, 280). These biomarkers are especially useful because they can reflect both tumor-intrinsic and immune-microenvironmental states. Transporter upregulation may indicate aggressive tumor nutrient acquisition, while enzyme expression in myeloid cells may point to ecosystem-level immune suppression. When interpreted in spatial and cellular context, such markers could help distinguish whether a tumor is dominated primarily by nutrient competition, suppressive metabolite signaling, or myeloid metabolic governance. This distinction may be important for selecting among different therapeutic strategies. However, expression alone is unlikely to be sufficient. Because amino acid pathways are regulated by flux, localization, and intercellular exchange, enzyme or transporter abundance should ideally be integrated with other data types. Even so, these markers provide a practical starting point for patient stratification and translational study design.

### 8.2 Metabolite-based immune monitoring

Beyond gene or protein expression, amino acid-derived metabolites themselves may serve as informative indicators of tumor immune status. Metabolite ratios and pathway-associated biochemical patterns have the potential to capture the integrated output of tumor metabolism, stromal regulation, and immune cell activity. This is especially valuable because metabolite levels may reflect actual pathway engagement more directly than static expression readouts (8, 230). Such monitoring could be useful in several ways. Baseline metabolite states may help identify tumors with dominant suppressive metabolic programs, while dynamic changes during treatment might reveal whether therapy is successfully reconditioning the tumor microenvironment. Metabolite-based approaches may also be able to track shifts in nutrient competition, suppressive signaling intensity, or emerging resistance mechanisms over time. If validated, they could support both patient selection and longitudinal treatment monitoring. The main challenge is interpretation. Metabolite levels are influenced by systemic physiology, tissue-specific context, and technical sampling variables. Therefore, metabolite-based immune monitoring will likely be most powerful when combined with spatial, cellular, and transcriptomic information rather than used in isolation.

### 8.3 Multi-omics decoding of amino acid metabolic niches

Because amino acid metabolic immune escape is inherently multicellular and spatially organized, multi-omics approaches are likely to be essential for fully decoding its biological and clinical significance. Single-cell transcriptomics, spatial transcriptomics, metabolomics, proteomics, and epigenomic profiling each capture different layers of the network described in this review (281–284). When integrated, these technologies can reveal which cells dominate amino acid uptake, where suppressive metabolites are most likely to accumulate, how immune cell states are metabolically conditioned, and which signaling circuits define resistant niches within tumors. This approach is particularly important for resolving heterogeneity. Bulk analyses may obscure localized metabolic barriers that are highly relevant to therapy response, whereas spatially resolved multi-omics can identify immunologically critical microregions in which nutrient competition and suppressive signaling converge. In addition, integrated profiling may uncover unexpected amino acid pathways or cell–cell interactions that would not emerge from candidate-based study designs. From a translational standpoint, multi-omics decoding offers a route toward precision immunometabolic oncology. Rather than applying broad metabolic therapies empirically, clinicians may eventually be able to identify the specific amino acid metabolic architecture of an individual tumor and choose interventions accordingly. This vision remains aspirational, but it represents one of the most promising future directions in the field.

## 9 Future perspectives

Future research should more clearly move from single-pathway descriptions toward an integrated understanding of amino acid metabolic networks in tumor immune escape. Although individual axes such as tryptophan–kynurenine, glutamine, arginine, methionine, and asparagine metabolism have provided important mechanistic insights, these pathways rarely operate in isolation. Instead, they are coordinated across tumor cells, myeloid populations, regulatory T cells, effector lymphocytes, stromal cells, endothelial compartments, and microbiota-related inputs (90, 285). A major priority is therefore to define how nutrient competition, metabolite signaling, epigenetic regulation, and immune-cell state remodeling interact as a network. Such studies should determine whether specific amino acid axes act redundantly, synergistically, or sequentially during tumor progression and immunotherapy resistance. This network-level perspective will be essential for identifying dominant metabolic nodes rather than treating all amino acid pathways as equivalent targets.

A second priority is the integration of multi-omics technologies to decode amino acid metabolic niches with cellular and spatial resolution. Bulk transcriptomic or metabolomic analyses may identify pathway activation but often fail to reveal which cell types drive amino acid consumption, where suppressive metabolites accumulate, and how immune-cell states are shaped by local metabolic constraints (92, 286). Future studies should combine single-cell transcriptomics, spatial transcriptomics, spatial metabolomics, proteomics, isotope tracing, and epigenomic profiling to map amino acid uptake, catabolism, metabolite exchange, and chromatin remodeling within intact tumor ecosystems (287, 288). This integrated approach could clarify how glutamine-deprived, kynurenine-rich, arginine-poor, or methionine-restricted niches correspond to CD8+ T-cell exhaustion, Treg stability, myeloid suppression, dendritic-cell dysfunction, and resistance to checkpoint blockade.

Third, the field needs robust metabolic biomarkers that can translate mechanistic knowledge into patient stratification and treatment monitoring. Candidate biomarkers may include amino acid transporters and enzymes, such as SLC1A5, SLC7A5, SLC43A2, GLS1, IDO1, TDO2, ARG1, MAT2A, and ASNS; metabolite ratios such as kynurenine/tryptophan or SAM/SAH; and immune-metabolic signatures reflecting T-cell exhaustion, Treg dominance, or suppressive myeloid polarization (289–291). Importantly, these biomarkers should not be evaluated only as static expression markers. They should be integrated with metabolite abundance, spatial localization, immune-cell composition, and treatment-induced dynamic changes. Longitudinal sampling before and during immunotherapy may help identify whether amino acid metabolic remodeling predicts primary resistance, adaptive resistance, or therapeutic response.

Finally, future therapeutic development should focus on rational combination strategies that target both amino acid metabolism and antitumor immunity. Because amino acid pathways are shared by tumor and immune cells, broad metabolic inhibition may impair protective immune responses. More precise approaches are needed, including tumor-selective metabolic targeting, myeloid reprogramming, Treg-limiting strategies, restoration of effector T-cell metabolic fitness, and combinations with PD-1/PD-L1 or CTLA-4 blockade. For example, IDO–kynurenine–AhR inhibition may be most effective in tumors with dominant tryptophan catabolism and tolerogenic myeloid programs, whereas glutamine-targeted strategies may require careful dosing to suppress tumor and myeloid metabolic dominance without damaging effector T cells (25, 292). Methionine- or one-carbon-directed interventions may need to be guided by epigenetic biomarkers and exhaustion-associated chromatin states. Therefore, the next stage of amino acid immunometabolism research should prioritize biomarker-guided, context-specific, and temporally optimized combination therapy.

Overall, the future of this field depends on connecting metabolic network biology with spatial multi-omics, clinically useful biomarkers, and rational immunotherapy combinations. This integrated framework will help transform amino acid metabolism from a descriptive feature of the tumor microenvironment into an actionable strategy for precision cancer immunotherapy.

## 10 Conclusion

Reprogrammed amino acid metabolism is now recognized as a central organizer of tumor immune escape rather than a secondary consequence of malignant growth. By reshaping nutrient availability, generating immunoregulatory metabolites, and rewiring immune cell states, tumors construct a metabolic microenvironment that selectively weakens antitumor immunity while preserving suppressive cellular programs. In this way, amino acid metabolism governs not only how tumors grow, but also how they evade immune destruction. A useful conceptual framework emerges from three interconnected layers. First, tumors and associated stromal and myeloid populations compete with immune cells for key amino acids, creating nutrient asymmetry within the tumor microenvironment. Second, amino acid catabolism produces metabolites that function as active signaling molecules, reinforcing immune tolerance, suppressive myeloid programming, and impaired lymphocyte function. Third, these pressures stabilize durable changes in immune cell identity, including CD8+ T-cell dysfunction, Treg persistence, macrophage-mediated suppression, and defective dendritic cell priming. Together, these processes form a metabolically governed immune ecosystem that promotes tumor persistence and contributes to immunotherapy resistance. This perspective has important translational implications. Therapeutic targeting of amino acid metabolism offers opportunities to weaken tumor metabolic dominance, disrupt suppressive signaling circuits, and improve responsiveness to checkpoint blockade and other immunotherapies. At the same time, the complexity, context dependence, and shared metabolic requirements of tumor and immune cells demand a careful and precise approach. Future progress will depend on biomarker-guided patient stratification, network-aware combination strategies, and integrated multi-omics approaches capable of resolving amino acid metabolic niches *in vivo*. Overall, amino acid metabolism provides a powerful framework for understanding the metabolic logic of tumor immune escape. Moving from nutrient competition to therapeutic targeting, this field is poised to reshape how cancer immunotherapy is conceptualized and, potentially, how it is practiced.
